# First descriptions of copepodid stages, sexual dimorphism and intraspecific variability of *Mesocletodes* Sars, 1909 (Copepoda, Harpacticoida, Argestidae), including the description of a new species with broad abyssal distribution

**DOI:** 10.3897/zookeys.96.1496

**Published:** 2011-05-10

**Authors:** Lena Menzel

**Affiliations:** Senckenberg am Meer Wilhelmshaven, Abt. DZMB, Südstrand 44, D- 26382 Wilhelmshaven, Germany

**Keywords:** ANDEEP, CROZEX, DIVA, Great Meteor Bank, meiofauna, *Mesocletodes elmari* sp. n., NODINAUT, ontogeny, Porcupine, Abyssal Plain

## Abstract

*Mesocletodes* Sars, 1909a encompasses 37 species to date. Initial evidence on intraspecific variability and sexual dimorphism has been verified for 77 specimens of *Mesocletodes elmari* sp. n. from various deep-sea regions, and ontogenetic development has been traced for the first time. Apomorphies are a strong spinule-like pinna on the mx seta that is fused to the basis, P2–P4 exp3 proximal outer seta lost, P1–P4 enp2 extremely elongated, furcal rami elongated, female body of prickly appearance, female P2–P4 enp2 proximal inner seta lost. Intraspecific variability involves spinulation, ornamentation and size of the body and setation and spinulation of pereiopods. Sexually dimorphic modifications of adult females include prickly appearance of the body, P1 enp exceeds exp in length, P1 coxa externally broadened, seta of basis arising from prominent protrusion, hyaline frills of body somites ornate. Sexual dimorphism in adult males is expressed in smaller body size, haplocer A1, 2 inner setae on P2–P4 enp2 and on P5 exp, P5 basendopodal lobe with 2 setae. Some modifications allow sexing of copepodid stages. The female A1 is fully developed in CV, the male A1 undergoes extensive modifications at the last molt. P1–P4 are fully developed in CV. *Mesocletodes faroerensis* and *Mesocletodes thielei* lack apomorphies of *Mesocletodes* and are excluded.

## Introduction

**Figure 1.**
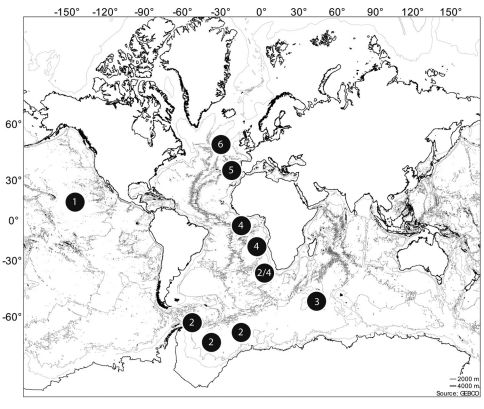
Positions of the sampled stations containing the species studied. **1** NODINAUT **2** ANDEEP **3** CROZEX **4** DIVA **5** GMB **6** PAP.

Expeditions to the Southeast Atlantic (DIVA-1 [Balzer et al. 2006], DIVA-2 [Türkay and Pätzold 2009] and part of ANDEEP III [Fahrbach 2006]), the Southern Ocean (ANDEEP I and II [Fütterer et al. 2003]), the South Indian Ocean (CROZEX [Pollard and Sanders 2006]), the central Pacific (NODINAUT [Galéron and Fabri 2004], the North Atlantic (Porcupine Abyssal Plain, PAP [see Kalogeropoulou et al. 2010 for summary] and the Great Meteor Bank [Pfannkuche et al. 2000]) (Fig. 1) provided numerous specimens of the genus *Mesocletodes* Sars, 1909a. Belonging to the family of Argestidae Por, 1986a, *Mesocletodes* is considered to be a typical and primarily deep-water dwelling taxon (compare overview in George 2004 and George 2008). The total number of *Mesocletodes* in deep-sea samples amounts to almost 50% of all Argestidae Por, which in turn form one of the most abundant taxa of harpacticoid copepods therein. Due to the high frequency in deep-sea samples and conspicuous morphological characters, *Mesocletodes* is informative for chorological, faunistic and biogeographic research. The number of specimens as well as species diversity are substantial, but species are well discernible.

*Mesocletodes* nowadays comprises 36 species (Menzel and George 2009; Wells 2007). All allied species show characteristic morphological features that allow rapid recognition in metazoan meiofauna samples: body of cylindrical shape, A1 segment 2 with conspicuous protrusion bearing a strong seta, md gnathobase with broad grinding face, P1 exp2 without inner seta, P1 exp3 without proximal outer spine, spines of this segment with subterminal tubular extensions, P2–P4 exp1 without inner seta, P2–P4 enps at most 2-segmented, telson square in dorsal and ventral view and furcal rami long and slender (cf. Menzel and George 2009).

The sex ratio of harpacticoid copepods in the deep sea is strongly biased towards females (e.g. Shimanaga et al. 2009; Shimanaga and Shirayama 2003; Thistle and Eckman 1990) and it is very difficult or nearly impossible to connect males and females of some species (e.g. Menzel and George 2009; Seifried and Veit-Köhler 2010; Vasconcelos et al. 2009; Willen 2006; Willen 2009; Willen and Dittmar 2009), indicating extremely poecilandric populations (Por 1986b). Concerning Argestidae, males could be connected to females for *Eurycletodes* Sars, 1909b, *Argestes* Sars, 1910, and *Hypalocletodes* Por, 1967 (cf. original descriptions). Since the establishment of *Mesocletodes* early in the 20th century (Sars, 1909a), this has been possible only for two species plus the herein described species. For 32 species of this genus only females are known, while exclusively males are known for two species.

Most of the species descriptions of *Mesocletodes* are based on few adult specimens (29 descriptions contain one to five type specimens, three descriptions are based on six to ten specimens, four descriptions are based on 11 to 16 specimens). Thus, neither intraspecific variability nor the process of ontogenetic development is reported for any species of *Mesocletodes*. Expeditions during the DIVA and ANDEEP campaigns yielded 54 out of 66 adults of *Mesocletodes elmari* sp. n. (more than 80%). The comparatively high frequency of specimens is probably explicable by the greater sampling effort in contrast to the CROZEX, NODINAUT, OASIS expeditions and sampling at the PAP as well as during previous campaigns. Repeated multicorer sampling of the same station (Martínez Arbizu and Schminke 2005; Rose et al. 2005) greatly enhances, for the first time, the opportunity of finding the same species again in one station or region. This implies that more specimens of one species are available, making investigations on intraspecific variability, specification of sexually dimorphic modifications and retracing of the ontogenetic development possible for the first time (cf. George 2008).

The aim of this publication is to convey an initial impression of the extent of sexually dimorphic modifications, ontogeny and intraspecific variability for the genus *Mesocletodes*, using *Mesocletodes elmari* sp. n. as an example.

## Material and methods

Sediment samples were taken with a multicorer (Barnett et al. 1984) in different oceanic regions: Southeast Atlantic (DIVA-1, DIVA-2 and part of ANDEEP III), Southern Ocean (ANDEEP I and II), South Indian Ocean (CROZEX), central Pacific (NODINAUT), North Atlantic (PAP and Great Meteor Bank) (Fig. 1, Table 1). Adult Harpacticoida were extracted from all samples, whereas copepodid stages are only available from the campaigns DIVA-1, DIVA-2 and ANDEEP.

**Table 1.** Specimens of *Mesocletodes elmari* sp. n. discovered, containing information on gender, number of eggs, ontogenetic stage, body length and remarks on intraspecific variability. f = female; m = male.

	Project	Expedition	Station	gender	number of eggs	ontogenetic stage	body length [mm]	remarks on intraspecific variability
	ANDEEP I	PS61	43/4-6	f		adult	0.70	
	ANDEEP I	PS61	46/4-1	f	ca. 15	adult	0.82	
	ANDEEP I	PS61	46/4-5	f		adult	0.63	P2 enp2 with outer seta
	ANDEEP I	PS61	46/4-8	f		adult	0.70	hyaline frill not ornate
	ANDEEP I	PS61	46/6-5	m		CV	0.50	
	ANDEEP I	PS61	46/6-3	f		adult	0.80	P2-P4 enp2 with outer seta
	ANDEEP I	PS61	46/6-3	f		adult	0.87	
	ANDEEP I	PS61	129/5-4	f	4	adult	0.78	
	ANDEEP II	PS61	131/11-A	f	4? 20?	adult	0.62	
	ANDEEP II	PS61	138/11-4	f		adult	0.71	
Paratype 6	ANDEEP II	PS61	138/11-4			CIII	0.47	
	CROZEX	D300	15773/31	f	11	adult	0.80	
	CROZEX	D300	15775/32	f		adult	0.92	FR: setular tuft near seta VII
	CROZEX	D300	15775/36	m		adult	0.43	
	DIVA-1	M48/1	325/6-2	f		adult	?	FR lost
	DIVA-1	M48/1	342/2-1	f		adult	0.71	FR: setular tuft near the base
	DIVA-1	M48/1	346/1-3	m		adult	0.68	
	DIVA-1	M48/1	346/1-7	m		adult	0.54	
	DIVA-1	M48/1	346/2-3	f		adult	0.83	
	DIVA-1	M48/1	346/2-3	f	12	adult	0.87	
	DIVA-1	M48/1	346/2-8	f	17	adult	0.86	
	DIVA-1	M48/1	346/2-9	f	3?	adult	0.74	
	DIVA-1	M48/1	346/2-11	f		adult	0.82	FR: setular tuft near seta VII, P1-P4 enp shorter
	DIVA-1	M48/1	346/3-9	f		adult	0.91	
	DIVA-1	M48/1	346/4-10	f		adult	0.85	FR: setular tuft near seta VII, P1-P4 enp shorter
	DIVA-1	M48/1	346/5-3	f		adult	0.80	hyaline frill not ornate
	DIVA-1	M48/1	346/5-9	f		adult	0.82	
	DIVA-1	M48/1	346/5-10	f	10	adult	0.66	
	DIVA-1	M48/1	346/6-2	f		adult	0.82	
	DIVA-1	M48/1	346/7-7	f		adult	0.85	
	DIVA-1	M48/1	346/7-8	f		adult	0.79	
	DIVA-1	M48/1	346/7-10	f		adult	0.78	
	DIVA-1	M48/1	346/7-10	f		CV	0.75	
Paratype 4	DIVA-1	M48/1	346/7-10	m		CV	0.59	
	DIVA-1	M48/1	346/8-3	f		adult	0.86	
Paratype 2	DIVA-2	M63/2	CAPE/35-7	f		adult	0.90	FR: setular tuft near seta VII
	DIVA-2	M63/2	CAPE/36-10	f	3	adult	0.57	
	DIVA-2	M63/2	CAPE/36-11	f		adult	0.66	
	DIVA-2	M63/2	CAPE/36-11	m		adult	0.40	
	DIVA-2	M63/2	CAPE/36-11	m		CV	0.40	FR: setular tuft near the base, P2-P4 enp2 with only 1 inner seta
	DIVA-2	M63/2	GUINEA E/56-5	m		adult	0.53	
	DIVA-2	M63/2	GUINEA E/57-1	f	2?	adult	0.70	FR: setular tuft near the base
	DIVA-2	M63/2	GUINEA E/57-8	f		adult	0.67	
	DIVA-2	M63/2	GUINEA E/58-10	f	7	adult	0.75	
	DIVA-2	M63/2	GUINEA E/58-12	f	5	adult	0.63	
	DIVA-2	M63/2	GUINEA E/59-10	f		adult	0.64	
	DIVA-2	M63/2	GUINEA E/59-12	m		adult	0.51	
	DIVA-2	M63/2	GUINEA E/61-4	m		adult	0.50	
	DIVA-2	M63/2	GUINEA E/62-6	f		adult	0.81	
	DIVA-2	M63/2	GUINEA E/62-6	m		adult	0.53	
	DIVA-2	M63/2	GUINEA W A/74-4	f		adult	0.78	hyaline frill not ornate, FR longer
Holotype	DIVA-2	M63/2	GUINEA W A/75-7	f		adult	0.78	
	DIVA-2	M63/2	GUINEA W A/76-6	f	7	adult	0.80	
	DIVA-2	M63/2	GUINEA W A/78-6	f		adult	?	FR lost
Paratype 1	DIVA-2	M63/2	GUINEA W A/78-7	m		adult	0.42	
	DIVA-2	M63/2	GUINEA W A/79-4	f		adult	?	Abdomen lost
Paratype 3	DIVA-2	M63/2	GUINEA W A/79-4	f		CV	0.55	P1 0-0 0,1,1, P2 0-0 0,2,0
	DIVA-2	M63/2	GUINEA W B/95-10	m		adult	0.43	
	DIVA-2	M63/2	GUINEA W B/96-8	f		adult	0.80	FR: setular tuft near the base, hyaline frill not ornate, FR longer
	DIVA-2	M63/2	GUINEA W B/97-6	f		adult	0.87	
	DIVA-2	M63/2	GUINEA W B/97-7	m		CIV	0.52	FR: setular tuft near the base, P1–P4 enp2 with outer seta
	DIVA-2	M63/2	GUINEA W B/97-7	m		CIV	0.49	FR: setular tuft near the base, P1–P4 enp2 with outer seta
	DIVA-2	M63/2	GUINEA W B/97-7	m		CIV	0.64	FR: setular tuft near the base, P1–P4 enp2 with outer seta
	DIVA-2	M63/2	GUINEA W B/97-7	m		CIV	0.47	FR: setular tuft near the base, P1–P4 enp2 with outer seta
Paratype 5	DIVA-2	M63/2	GUINEA W B/97-7	m		CIV	0.43	FR: setular tuft near the base, P1–P4 enp2 with outer seta
	DIVA-2	M63/2	GUINEA W B/99-10	f	10	adult	0.71	FR: setular tuft near the base, P2-P4 enp2 with outer seta, denticulation of hyaline frill more dense
	DIVA-2	M63/2	GUINEA W B/100-6	f		adult	0.87	
	DIVA-2	M63/2	GUINEA W B/100-7	f	5	adult	0.90	
	GMB	M42/3	505	f		adult	0.61	
	GMB	M42/3	505	f		adult	0.61	
	GMB	M42/3	566	f		adult	0.76	FR: setular tuft near the base
	NODINAUT		1599/7-2	f	4	adult	0.65	
	NODINAUT		1599/7-3	f		adult	0.66	
	NODINAUT		1602/10-7	f	5	adult	0.69	
	NODINAUT		1602/10-8	f	5	adult	0.67	
	NODINAUT		1603/11-1	f		adult	1.06	
	PAP		Mar 1997/13077-12	f	1?	adult	0.63	FR: setular tuft near the base

Altogether 77 specimens (56 adult females, 10 adult males, 2 CV females, 3 CV males, 5 CIV males and 1 CIII) were found. The type material of *Mesocletodes elmari* sp. n. consists of 7 specimens (2 females plus 1 each of the other discovered stages). The type material was deposited in the collection of the Senckenberg Forschungsinstitut und Naturmuseum Frankfurt (Germany). The remaining 70 specimens are mounted on slides and kept in the collection of the DZMB in Wilhelmshaven (Germany).

The material was mounted on separate slides using glycerol as the embedding medium. Identification at the species level and drawings were carried out using a Leica microscope DM2500 equipped with a camera lucida and interference contrast with a maximum magnification of 1600x.

The CLSM photograph of a Congo-red stained female was taken with a Leica TCS SP5 mounted in a Leica DM5000. Preparations and settings were made according to Michels and Büntzow (2010).

Abbreviations used in the present paper are: A1 (antennula), A2 (antenna), aes (aesthetasc), benp (baseoendopod), CI–CV (copepodid stages 1–5), cphth (cephalothorax), enp (endopod), exp (exopod), FR (furcal rami), GF (genital field), md (mandibula), mx (maxilla), mxl (maxillula), mxp (maxilliped), P1–P6 (pereiopods 1–6), STE (Subterminal Tubular Extension, according to Huys 1996).

I could examine other material for comparison: Type material of *Mesocletodes parabodini* Schriever, 1983, (1 dissected female, ZMK Cop. No. 1319). *Mesocletodes farauni* Por, 1967 (1 female, dissected, HUJ Cop no. 69 plus one additional specimen), *Mesocletodes glaber* Por, 1964a (1 female, dissected, HUJ Cop no. 33) and *Mesocletodes monensis* (Thompson, 1893) (3 females, dissected, on one slide each, HUJ Cop no. 63, 93, 138).

## Taxonomy

**Argestidae Por, 1986a**

### Mesocletodes

Sars, 1909a

http://species-id.net/wiki/Mesocletodes

#### Type species:

*Mesocletodes irrasus* (T. and A. Scott, 1894), (described as *Cletodes irrasa*)

#### Additional species:

*Mesocletodes* contains 37 species (Boxshall and Halsey 2004; Menzel and George 2009; Wells 2007), including the type species and the herein described new species: *Mesocletodes abyssicola* (T. and A. Scott, 1901), *Mesocletodes angolaensis* Menzel and George, 2009, *Mesocletodes bathybia* Por, 1964b, *Mesocletodes bicornis* Menzel and George, 2009, *Mesocletodes brevifurca* Lang, 1936, *Mesocletodes dolichurus* Smirnov, 1946, *Mesocletodes dorsiprocessus* Menzel and George, 2009, *Mesocletodes katharinae* Soyer, 1964, *Mesocletodes meteorensis* Menzel and George, 2009, *Mesocletodes monensis*, *Mesocletodes opoteros* Por, 1986b, *Mesocletodes quadrispinosa* Schriever, 1985, *Mesocletodes robustus* Por, 1965, *Mesocletodes soyeri* Bodin, 1968, *Mesocletodes ameliae* Soyer, 1975, *Mesocletodes arenicola* Noodt, 1952, *Mesocletodes bodini* Soyer, 1975, *Mesocletodes carpinei* Soyer, 1975, *Mesocletodes commixtus* Coull, 1973, *Mesocletodes duosetosus* Schriever, 1985, *Mesocletodes farauni*, *Mesocletodes faroerensis* Schriever, 1985, *Mesocletodes fladensis* Wells, 1965, *Mesocletodes glaber*, *Mesocletodes guillei* Soyer, 1964, *Mesocletodes inermis* Sars, 1921, *Mesocletodes irrasus*, *Mesocletodes kunzi* Schriever, 1985, *Mesocletodes langi* Smirnov, 1946, *Mesocletodes makarovi* Smirnov, 1946, *Mesocletodes parabodini* Schriever, 1983, *Mesocletodes parirrasus* Becker, Noodt & Schriever, 1979, *Mesocletodes sarsi* Becker, Noodt & Schriever, 1979, *Mesocletodes thieli* Schriever, 1985, *Mesocletodes trisetosa* Schriever, 1983, *Mesocletodes variabilis* Schriever, 1983, *Mesocletodes elmari* sp. n.

#### Generic diagnosis

(amended from Sars 1909a and Soyer 1964): Body of cylindric form, distal edge of body somites with many spinules close to hyaline frill, integument thin and flexible. Cphth not longer than first 2 free prosomites together, rostrum small. Telson as long as 2 last urosomites together, square from lateral and ventral view. FR longer than wide, seta VII in the proximal third. A1 6–8-segmented in females, second segment with strong protrusion bearing 1 strong bipinnate seta pointing backwards. A2 with basis or allobasis, without abexopodal seta, exp at most 1-segmented with at most 2 setae. Md palpus with at most 1-segmented exp and enp, blades of gnathobase forming broad grinding face. Mxl palp enp segment incorporated into basis or absent, exp segment present, incorporated into basis or absent. Mx proximal endite with 1 seta. Mxp prehensile, with strong claw distally. P1–P4 exps 3-segmented, of P1 small, of P2–P4 long and slender. P1 exp3 with 4 setal elements, spines with STEs. P1–P4 enps at most biarticulate. P5 exp longer than wide, endopodal lobe barely protruding. 1 egg sack with 2–40 eggs.

#### Mesocletodes elmari sp. n.

urn:lsid:zoobank.org:act:8056C1B4-FC83-4410-BE24-7F495F056509

http://species-id.net/wiki/Mesocletodes_elmari

Figs 2 14

##### Etymology:

The name is dedicated to the author’s father, Elmar Menzel.

##### Locus typicus:

Guinea Basin, RV “Meteor“, Cruise M63/2 (DIVA-2), station 75/7 (0°50.0'N, 5°35.0'W, 5139m), March 19, 2005.

##### Type material:

7 individuals Holotype: 1 female, dissected, mounted on 17 slides, coll. no. SMF 37012/1–17, RV “Meteor“, Cruise M63/2 (DIVA-2) at station 75/7 (0°50.0'N, 05°35.0'W, 5139m), March 19, 2005.

Paratypes: Paratype 1 (Allotype): 1 male, dissected, mounted on 9 slides, coll. no. SMF 37013/1–9, RV “Meteor“, Cruise M63/2 (DIVA-2) at station 78/7 (0°50.1'N, 05°35.1'W, 5136m), March 19, 2005.

Paratype 2: 1 female, mounted on 1 slide, coll. no. SMF 37014, RV “Meteor“, Cruise M63/2 (DIVA-2) at station 35/7 (28°6.8'S, 7°20.7'E, 5033m), March 03, 2005.

Paratype 3: 1 CV female dissected, mounted on 6 slides, coll. no. SMF 37015/1–6, RV “Meteor“, Cruise M63/2 (DIVA-2) at station 79/4 (0°50.0'N, 05°35.1'W, 5140m), March 19, 2005.

Paratype 4: 1 CV male dissected, mounted on 2 slides, coll. no. SMF 37016/1–2, RV “Meteor“, Cruise M48/1 (DIVA-1) at station 346-7/10 (16°17.0'S, 05°27.0'E, 5389m), July 27, 2000.

Paratype 5: 1 CIV male dissected, mounted on 8 slides, coll. no. SMF 37017/1–8, RV “Meteor“, Cruise M63/2 (DIVA-2) at station 97/7 (0°37.2'N, 06°28.1'W, 5168m), March 23, 2005.

Paratype 6: 1 CIII dissected, mounted on 7 slides, coll. no. SMF 37018/1–7, RV “Polarstern“, Cruise PS61 (ANT-XIX/4 (ANDEEP II)) at station 138-11/4 (62°58.03'S, 27°54.08'W, 4541m) March 18, 2002.

##### Description of adult female holotype.

**Figure 2.**
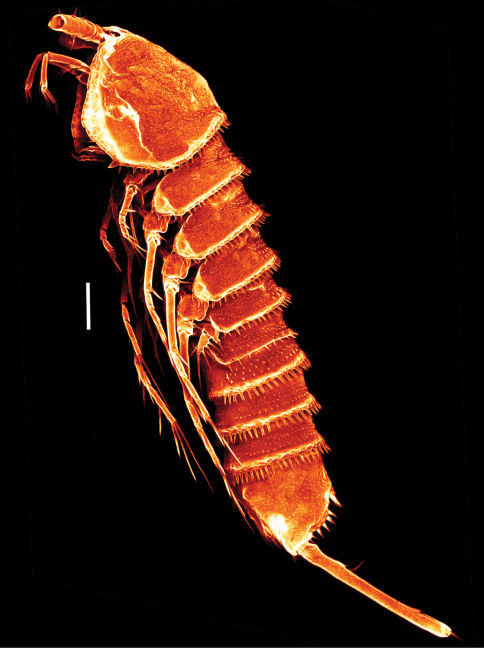
*Mesocletodes elmari* sp. n., adult female, paratype 2. CLSM photograph of a Congo-red stained specimen, lateral view. Scale bar: 100 µm

**Figure 3.**
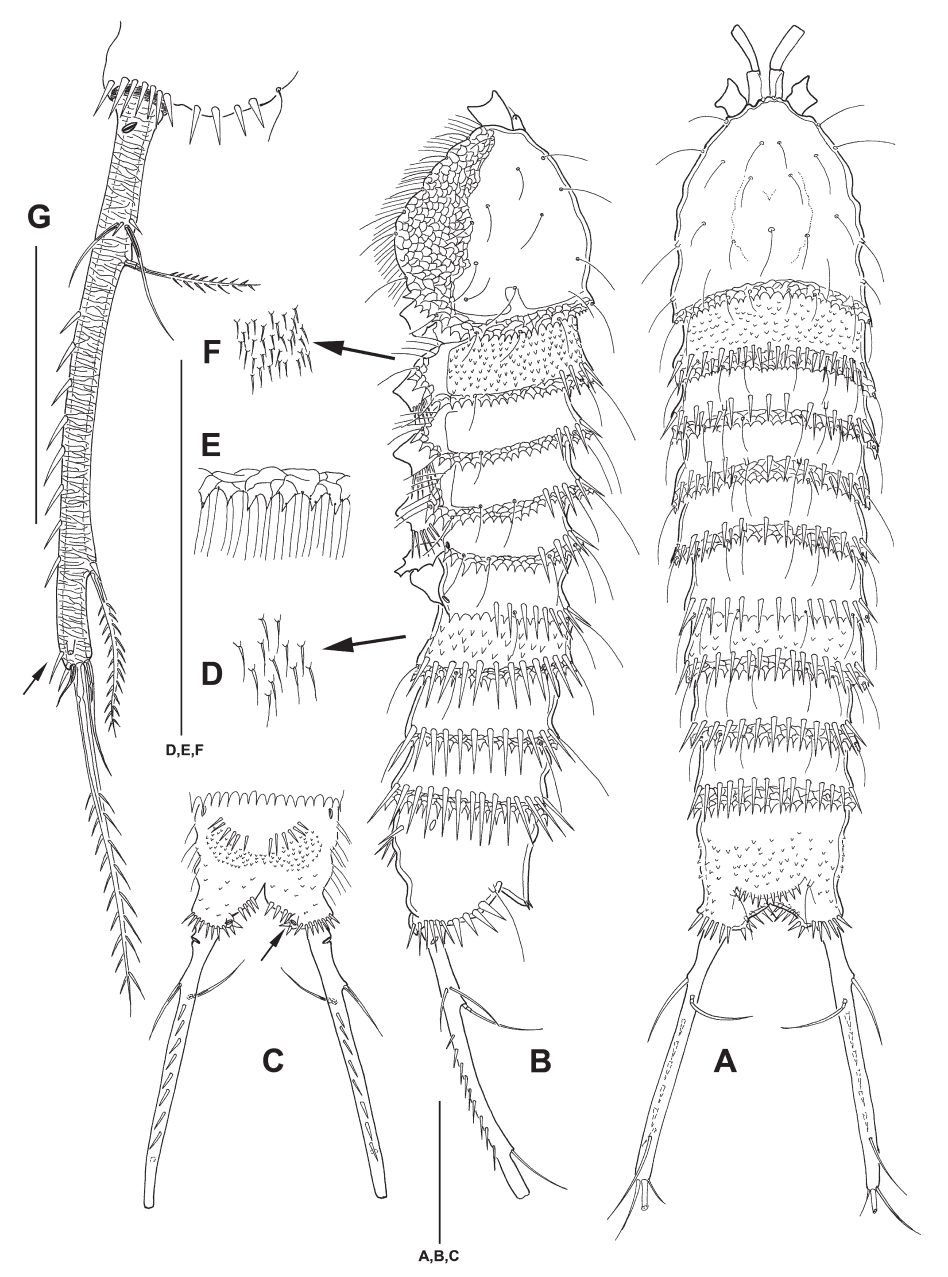
*Mesocletodes elmari* sp. n., adult female, holotype. **A** habitus dorsal view **B** habitus lateral view **C** telson ventral view, internal notch-like pores indicated by arrow **D** detail of urosomal setules **E** detail of hyaline frill **F** detail of prosomal setules **G** FR lateral view, tube pores indicated by arrow. Scale bars: **A–C**: 100 µm; **D–G**: 50 µm

**Figure 4.**
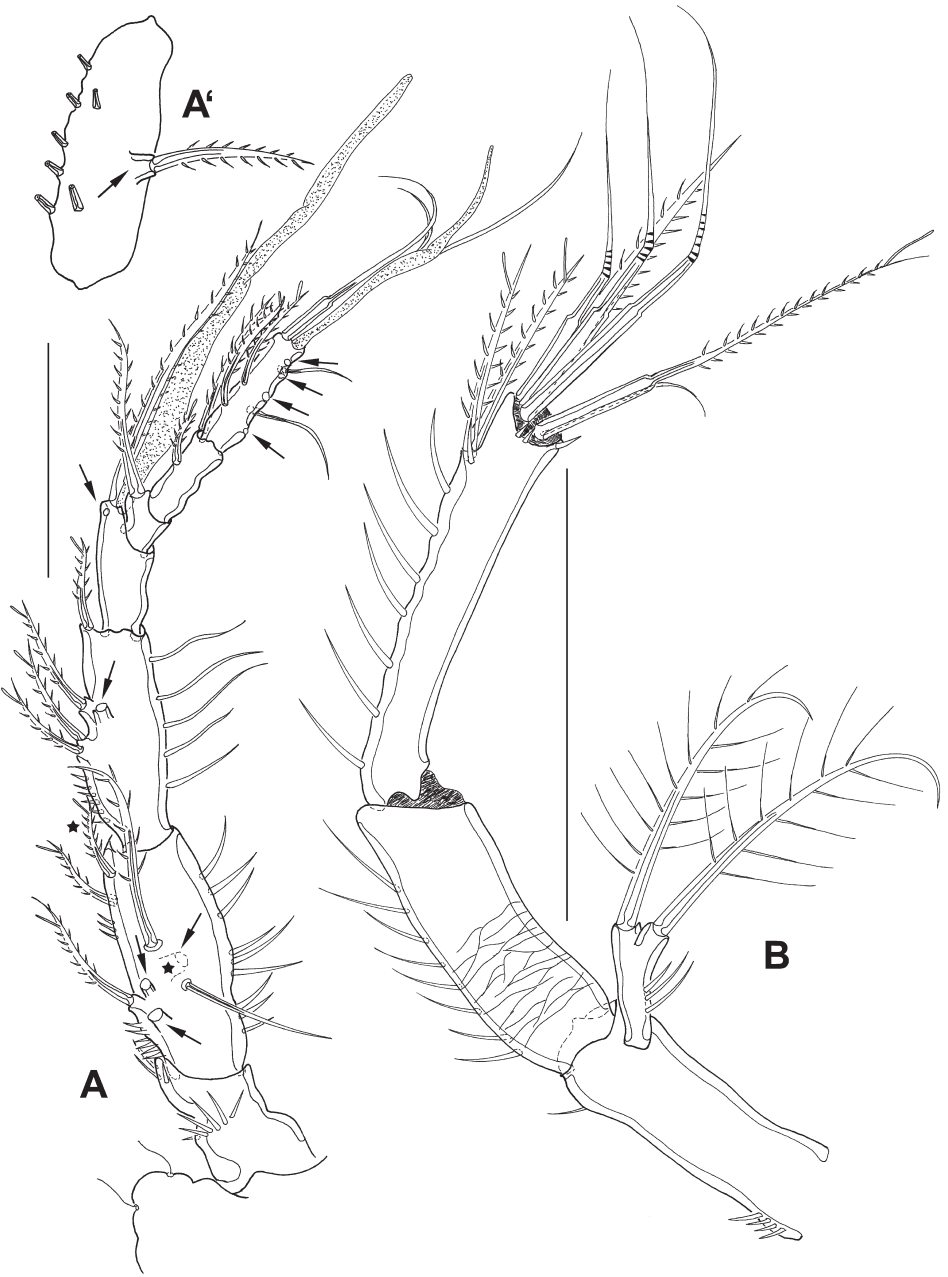
*Mesocletodes elmari* sp. n., adult female. **A** A1, holotype, dorsal view. Missing setae indicated by arrows. Asterisks mark the 2 setae presumably occurring in CV. **A’** second A1 segment, paratype 2, ventral view, arrow indicates characteristic protrusion with seta **B** A2 holotype. Scale bars: 50 μm

**Figure 5.**
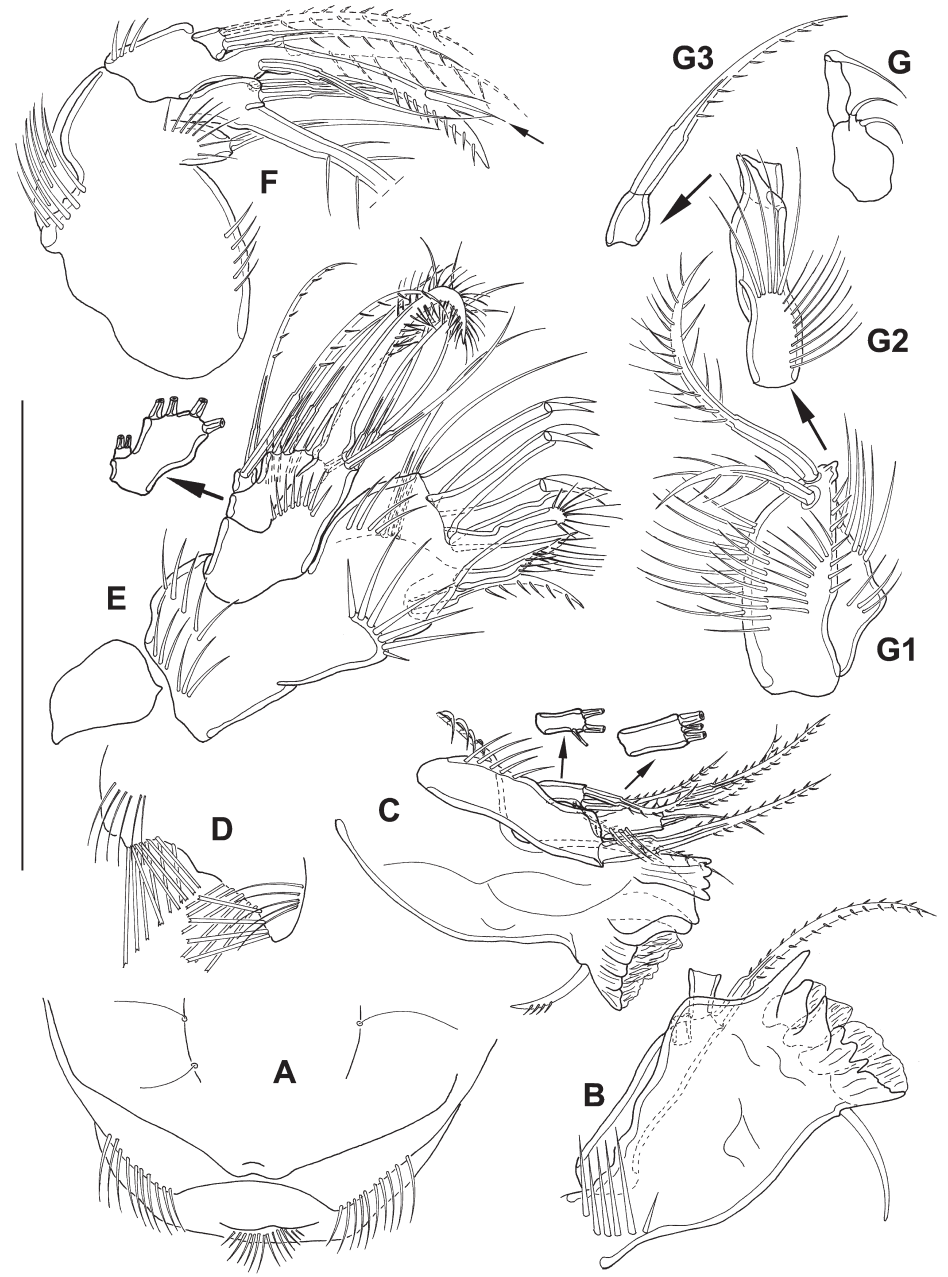
*Mesocletodes elmari* sp. n., adult female. **A** labrum, holotype **B+C** md, holotype, **D** paragnaths, holotype **E** mxl, holotype **F** mx, holotype, basal seta supplemented after counterpart, dash-depicted endopodal seta supplemented after paratype 2. Arrow dicates the peculiar spinulelike pinna **G** mxp, paratype 2, unfragmented, **G1–G3** mxp details, holotype. Scale bar: 50 µm

**Figure 6.**
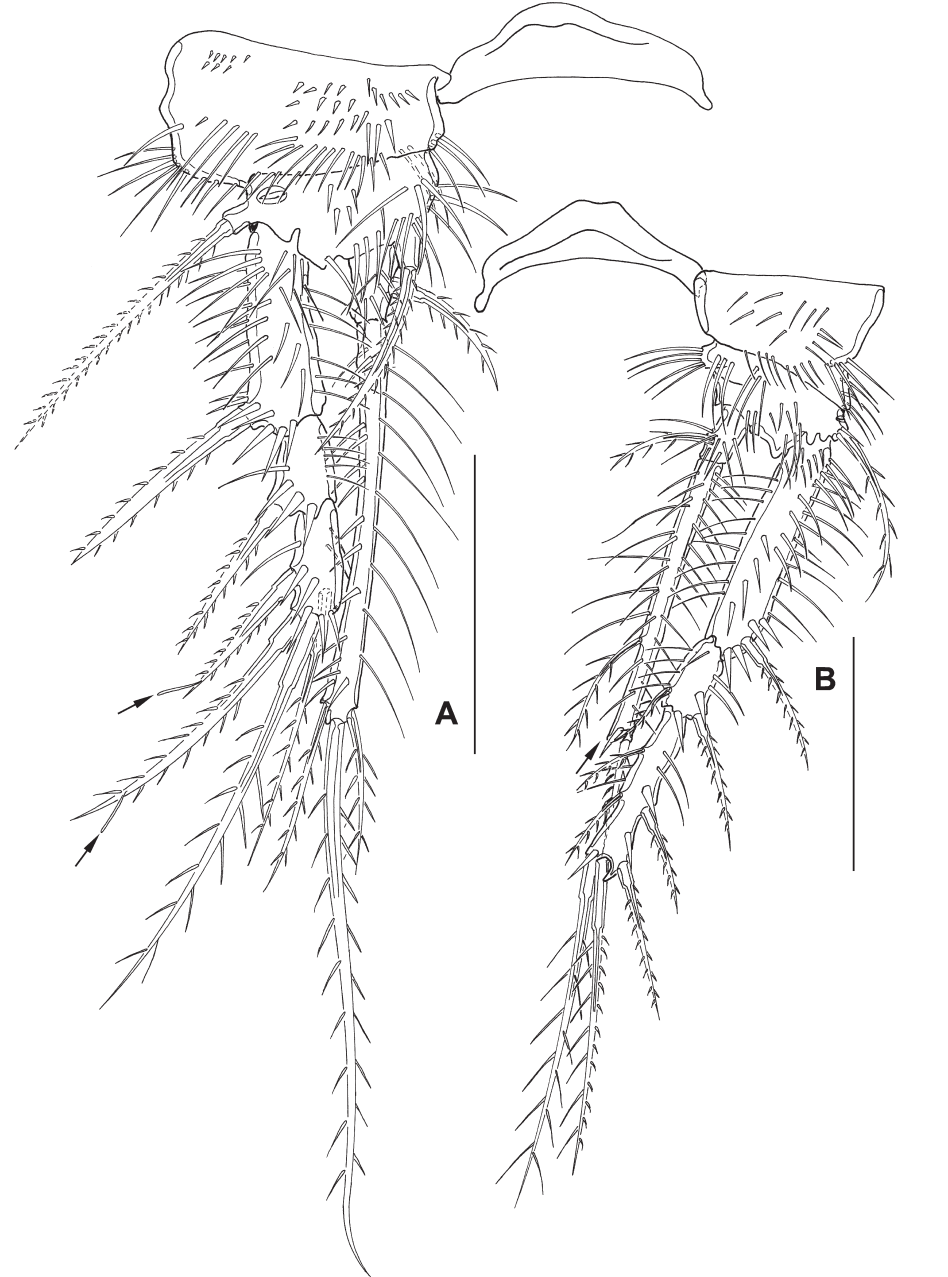
*Mesocletodes elmari* sp. n., adult female, holotype. **A** P1, tube pores indicated by arrows **B** P2. Scale bars: 50 µm

**Figure 7.**
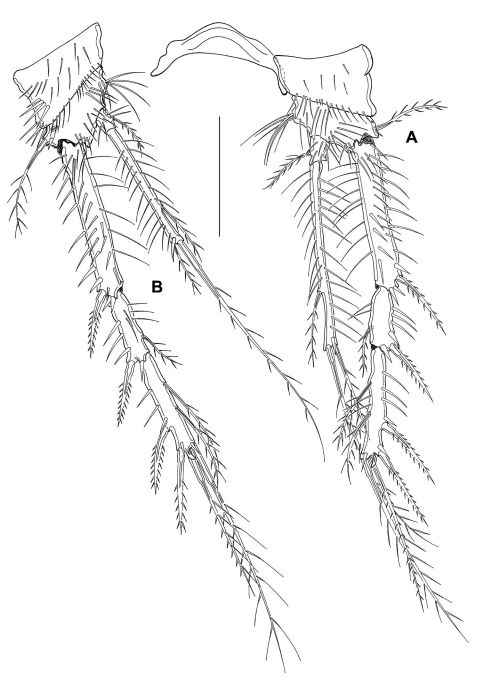
*Mesocletodes elmari* sp. n., adult female, holotype. **A** P3 **B** P4. Scale bar: 50 µm

**Figure 8.**
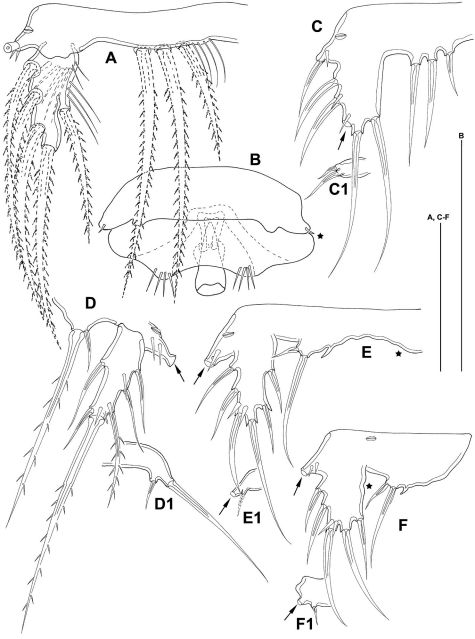
*Mesocletodes elmari* sp. n. **A** adult female holotype, P5, dorsal view **B** adult female holotype, GF, P6 indicated by asterisk **C** CV female paratype 3, P5 ventral view **C1** CV female paratype 3, P6 ventral view **D** adult male paratype 1, P5 ventral view **D1** adult male paratype 1, P6 ventral view **E** CV male paratype 4, P5 ventral view, asterisk on the right side of the endopodal lobe indicates where a cuticular protrusion analogous to the one on the left side can be expected **E1** CV male paratype 4, P6 ventral view **F** CIV male paratype 5, P5 ventral view, asterisk marks the inner depression on P5 exp **F1** CIV male paratype 5, P6 ventral view. Missing setae indicated by arrows. Scale bars: 50 µm.

(Figs 2–8) Habitus (Figs 2 [paratype], 3 A – B) of cylindrical shape, no clear distinction between prosome and urosome. Body length including FR 0.78 mm. Distal margins of cphth, prosomites and urosomites with conspicuous coarsely ornate and denticulated hyaline frill with many setules (Fig. 3 E). Body with several remarkably long sensilla. Distal margins of prosomites with long spinules: only dorsally in prosomites and first urosomite, in urosomites also laterally and ventrally. Distal margin of last urosomite without sensilla. Rostrum not protruding, with 2 sensilla. Body of prickly appearance, caused by small protrusions bearing one setule each, protrusions in urosomites and telson coarser than in prosomites (Fig. 3 D, F). Notch-like pores ventrolaterally on P4 – P5 bearing somites. Genital double somite fused ventrally. Telson (Fig. 3 A–C) as long as 2 preceding urosomites together, almost square from lateral and dorsal view. Ventrally with 2 rows of 6 long spinules each and on the outer edges, close to hyaline frill of last urosomite. 1 ventral notch-like pore on each side at inner edge near insertion of FR. Operculum with several denticles (Fig. 3 A).

A1 (Fig. 4 A, A’) 7-segmented, reticulated as shown for proximal part of A2 enp1 (Fig. 4 B). Segments 4 and 7 with aes. Second segment of paratype 2 (A’) large, with 1 protrusion bearing 1 bipinnate seta (seta lost during preparation of holotype). Spines with STEs. First and second segment bear inner and outer spinules, third segment with outer spinules. Setal formula: 1: 0; 2: 8; 3: 5; 4: 2+aes; 5: 1; 6: 2; 7: 9+acrothek (=11+aes).

A2 (Fig. 4 B) with basis, reticulate ornamentation as shown for part of enp1. Exp 1-segmented, with 1 terminal and 1 subterminal seta. Enp 2-segmented, both segments with strong outer spinules. Enp2 with 2 bipinnate spines subterminally. 3 geniculate and 2 pinnate spines, and 1 naked seta terminally. Naked terminal seta fused basally to 1 outer pinnate spine. The innermost element is a reduced seta. Spines with STEs.

Labrum (Fig. 5 A) with 1 medial and 2 lateral rows of spinules, setules at oral surface.

Md (Fig. 5 B, C) gnathobase formed by 5 tooth-like projections: 1 dentate, 1 broad tooth, 3 strong teeth partly fused to broad grinding face. Strong seta close to grinding face. Md palpus 3-segmented, exp and enp articulated. 1 strong basal seta terminally, exp with 2 terminal and 1 subterminal setae, enp with 3 terminal setae.

Paragnaths (Fig. 5 D) on each side with 2 rows of traverse arranged brush-like setae orally and 1 row of long spinules at the surface.

Mxl (Fig. 5 E) praecoxal arthrite terminally with 6 strong elements: 3 hooks with 1 strong spinule each, 1 brushlike seta fused to arthrite and 2 unipinnate setae. Subterminally with another pinnate spine and 2 bare setae aborally. Coxa with 4 elements terminally: 1 strong seta fused to coxa and 3 bare setae. Basis with 2 bare setae. Enp incorporated into basis, with 2 bare setae, exp 1-segmented with 2 pinnate setae.

Mx (Fig. 5 F) syncoxa with 2 endites, the proximal one bearing 1 seta. Distal endite with 3 setae, the biggest one fused to segment. 2 strong setae fused to basis, distal one shows a suture, proximal one with 1 conspicuous strong spinule-like pinna (indicated by arrow in Fig. 5 F). Basis additionally with 1 bare seta. Enp 1-segmented, with 2 bipinnate setae of equal length (dash-depicted seta supplemented from paratype 2).

Mxp (Fig. 5 G, G1–G3) prehensile, syncoxa (Fig. 5 G1) slightly shorter than basis (proximal part of Fig. 5 G2), with 2 setae and several spinules. Basis slender, with spinules of different sizes. Enp 2-segmented. Enp1 (distal part of Fig. 5 G2) small, bare of setae. Enp2 (Fig. 5 G3) terminally fused to strongly pinnate claw, suture visible.

P1 (Fig. 6 A) with 3-segmented exp and 2-segmented enp. Intercoxal sclerite long and bow-like. Coxa 1/3 broader than basis, with several spinules on ventral margin. Basis with outer spine, outer pore, long inner spine ventrally oriented and several rows of spinules. Exp1 and exp2 without inner seta. Exp3 with 4 elements. Enp1 short, with strong inner spine inserted medially. Enp2 extremely long, surpassing exp in length, with 1 outer, 1 terminal and 1 inner seta. Enp2 with 1 peculiar spinule subterminally. For setal formula see Table 2.

**Table 2.** *Mesocletodes elmari* sp. n., setal formula of P1–P4 of adults and copepodid stages. Pereiopodal setation of CV female and CV male is analogous to adults. – = segment is missing

		exp1	exp2	exp3	enp1	enp2
P1	adult female	I-0	I-0	I,I1,1	0-1	1,1,1
	adult male	I-0	I-0	I,I1,1	0-1	0,1,1
	CIV male	I-0	2,I1,1	–	0-1	1,1,1
	CIII	I-0	2,I1,1		0-1	0,1,1
P2	adult female	I-0	I-1	II,I1,2	0-1	0,2,1
	adult male	I-0	I-1	II,I1,2	0-1	0,2,2
	CIV male	I-0	III,I1,3	–	0-1	1,2,1
	CIII	I-0	III,I1,2	–	0-1	0,2,1
P3	adult female	I-0	I-1	II,I1,2	0-1	0,2,1
	adult male	I-0	I-1	II,I1,2	0-1	0,2,2
	CIV male	I-0	III,I1,3	–	0-1	1,2,1
	CIII	I-0	II,I1,2	–	0-1	0,2,0
P4	adult female	I-0	I-1	II,I1,2	0-1	0,2,1
	adult male	I-0	I-1	II,I1,2	0-1	0,2,2
	CIV male	I-0	III,I1,3	–	0-1	1,2,1
	CIII	III,I1,0	–	–	0,2,0	–

P2–P4 (Figs 6 B, 7 A, B) with 3-segmented exps and 2-segmented enps. Intercoxal sclerites long and bow-like. Coxae little larger than bases. Bases twice as broad as long. Bases with outer spines, at inner margin with setular tufts. Outer margins of coxa with strong spinules, inner margins of coxa and basis with setules. Exp1 as long as exp2 and exp3 together. Exp1 without inner seta. Exp3 terminally with cuticular hooks. Enp1 short. Enp2 extremely long, decreasing in length from P2-P4, measured in relation to exp1. Enp2 with 1 strong, short spinule subterminally. Outer terminal seta of enp2 decreasing in length from P2–P4. Inner terminal seta in P2 enp2 lost during preparation (indicated by arrow in Fig. 6 B). Setation of exp and enp as in Table 2.

P5 (Fig. 8 A) benp with setophore with 2 spinules and 1 long bipinnate seta. Endopodal lobe not protruding, with 3 setae. Exp about 2 times as long as broad at base, bearing 3 outer, 1 terminal and 1 inner seta (dash-depicted setae supplemented from paratype 2).

P6 integrated into GF (Fig. 8 B), reduced to a fused opercular plate, armed with 1 short spine on each side (see asterisk in Fig. 8 B). GF with single aperture, accompanied by 1 row of spinules on each side.

FR (Fig. 3 G) long and slender, ornate, ventral spinules between seta VII and III. Approximately 13 times as long as broad (measured at base). Close to base ventrolaterally with 1 notch-like pore at external side (Fig. 3 G, C). Extremely elongated between setae VII and III. Seta I close to seta II. Seta VII triarticulate. Seta III located on dorsal side subterminally. Setae IV–VI located terminally. FR laterally with subterminal tube pore (see arrow in Fig. 3 G).

##### Description of adult male paratype

**Figure 9.**
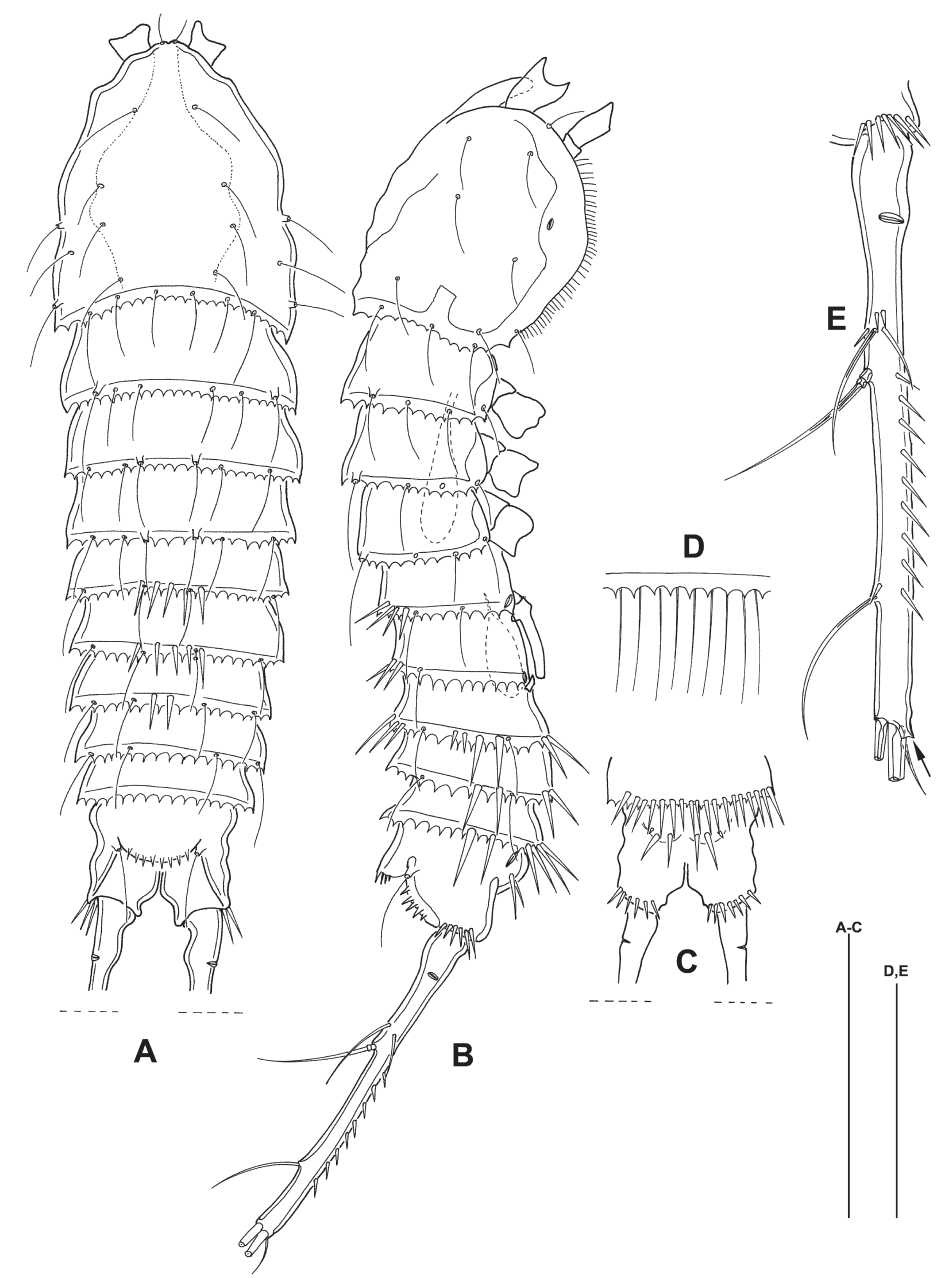
*Mesocletodes elmari* sp. n., adult male paratype 1. **A** habitus dorsal view **B** habitus lateral view **C** telson ventral view **D** detail of hyaline frill **E** FR, lateral view, arrow indicates terminal tube pore. Scale bars: **A–C** 100 µm, **D+E** 50 µm.

**Figure 10.**
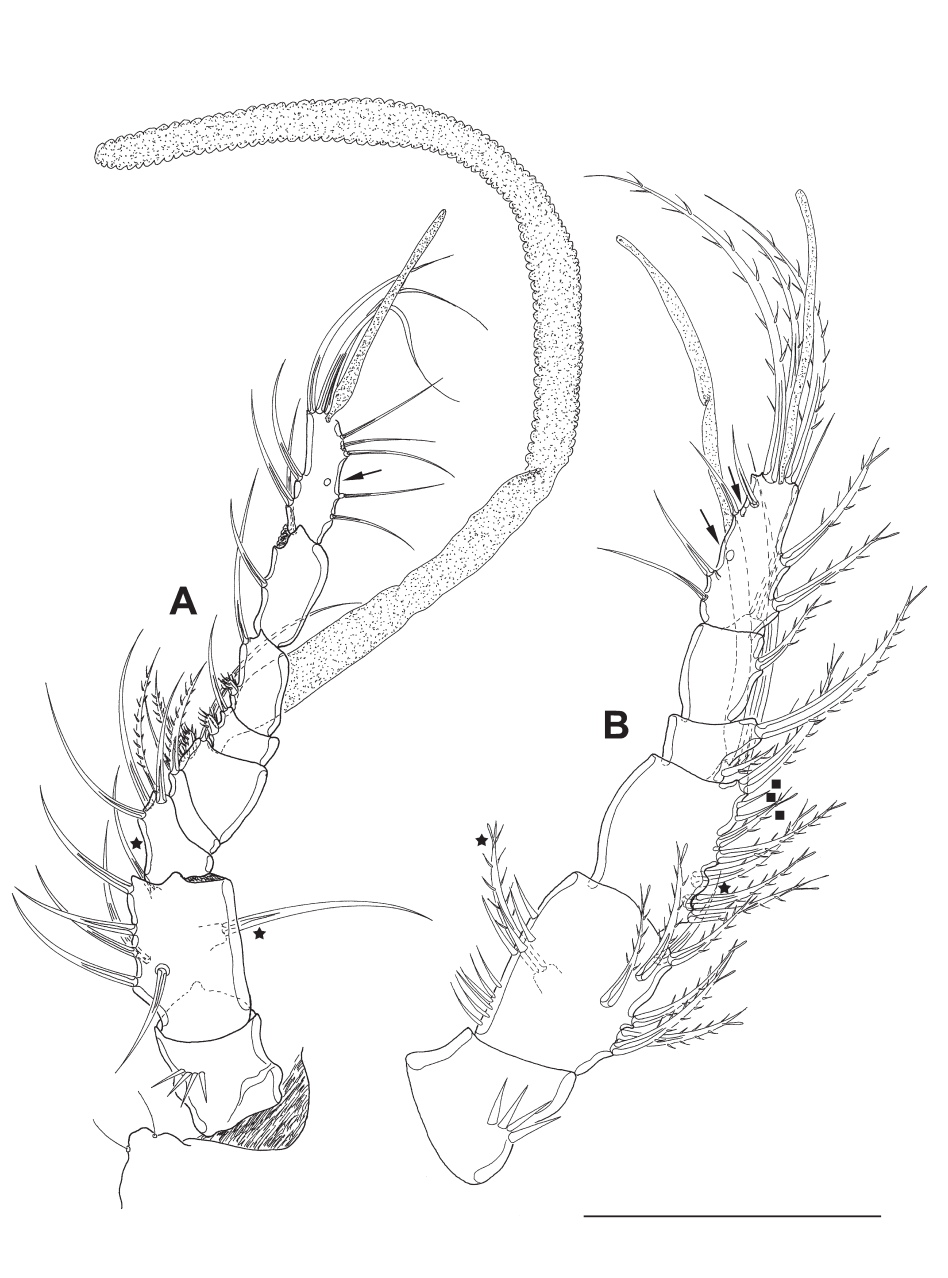
*Mesocletodes elmari* sp. n. **A** adult male paratype 1, A1 dorsal view **B** CV male paratype 4, A1 dorsal view, minute setae on third segment highlighted by solid squares. Asterisks mark the 2 setae occurring in CV. Missing setae indicated by arrows. Scale bar: 50 µm.

**Figure 11.**
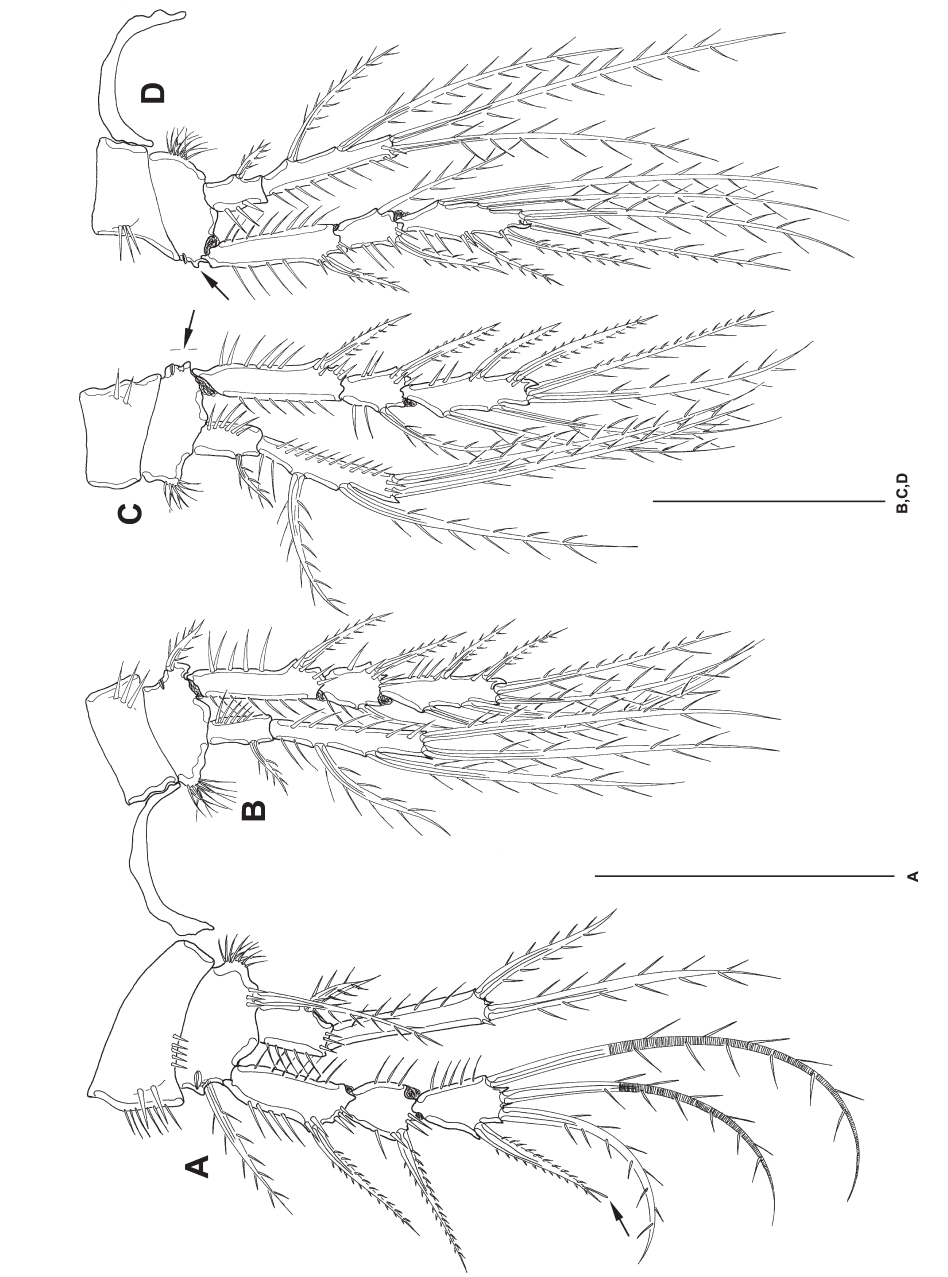
*Mesocletodes elmari* sp. n., adult male paratype 1. **A** P1 **B** P2 **C** P3 **D** P4. Missing setae indicated by arrows. Scale bars: 50 µm.

(Allotype) (Figs 8–11) The adult male corresponds to the adult female in all morphological characters unless deviations are mentioned below.

Habitus (Fig. 9 A, B) much smaller than adult female, body length including FR 0.40 mm. Body not of prickly appearance (Fig. 9 A–C), hyaline frill (Fig. 9 D) not ornate. Distal margins of first and second urosomites with long spinules dorsally, of third urosomite dorsally, laterally and ventrally, of last 2 urosomites only laterally and ventrally. With 2 spermatophores: first one inside first urosomite, second one inside second and third prosomite. Gut empty. FR (Fig. 9 E) as described for female.

A1 (Fig. 10 A) 9-segmented, haplocer. Segments 5 and 9 with aes. Second segment large, with 1 protrusion bearing 1 bare seta. Segments 5, 6 and 7 with modified setae. Setae of most segments bare. Setal formula: 1: 0; 2: 8; 3: 4; 4: 2; 5: 4+aes; 6: 2; 7: 2; 8: 2; 9: 9+acrothek (=11+aes).

A2, Md, Mxl, Mx and Mxp as described for adult female.

P1–P4 (Fig. 11 A–D) intercoxal sclerites, coxae, bases and segmentation of enp and exp as described for adult female, but with fewer spinules. P1 exp3 with 1 spine and 3 setae, the 2 innermost of wreathed appearance. P2–P4 inner exopodal setae long. P2–P4 enp2 with 2 long inner setae. Basal seta of P3 and P4 broken (indicated by arrow in Fig. 11 C, D). For setal formula see Table 2.

P5 (Fig. 8 D) with setophore (seta lost during dissection, see arrow in Fig. 8 D) with few spinules and 1 notch-like pore laterally. Endopodal lobe barely protruding, with 2 setae, outermost very short. Exp about twice as long as broad (measured at base), bearing 3 outer, 1 terminal and 2 inner setae.

P6 (Fig. 8 D1) with 2 setae.

##### Description of copepodid stages

**Figure 12.**
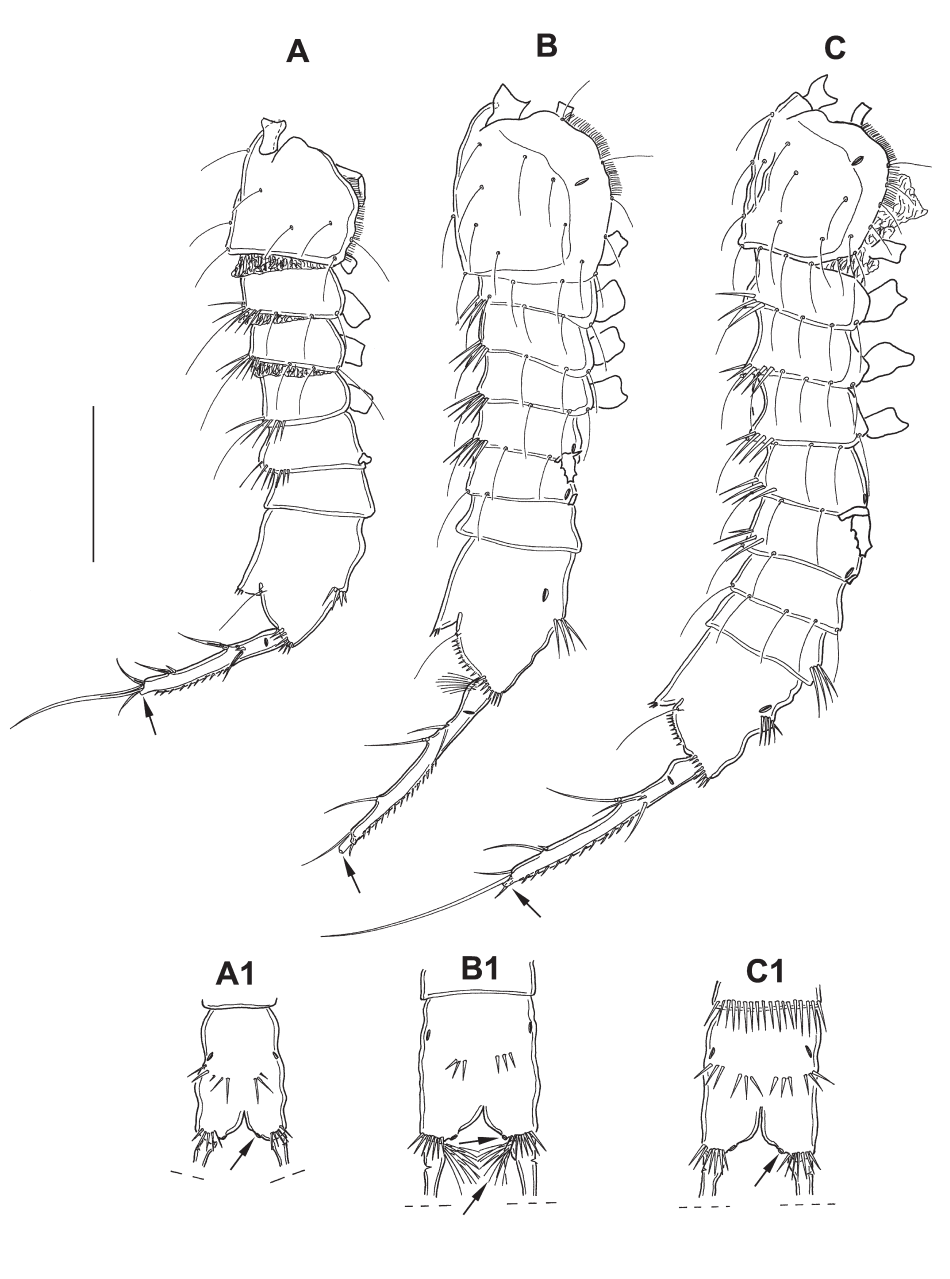
*Mesocletodes elmari* sp. n. **A** CIII paratype 6, habitus lateral view, terminal TP on FR indicated by arrow **A1** CIII paratype 6, telson ventral view, internal notch-like pores indicated by arrow **B** CIV male paratype 5, habitus lateral view, terminal TP on FR indicated by arrow **B1** CIV male paratype 5, telson ventral view, internal notch-like pores and setular tuft on FR indicated by arrows **C** CV female paratype 3, habitus lateral view, terminal TP on FR indicated by arrow **C1** CV female paratype 3, telson ventral view, internal notch-like pores indicated by arrow. Scale bar: 100 µm.

**Figure 13.**
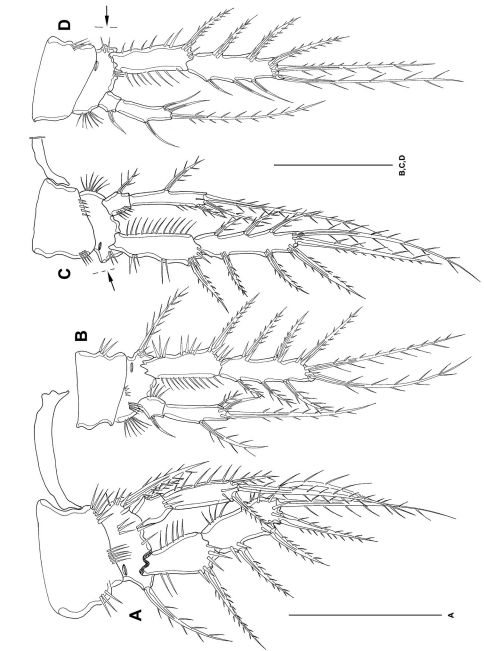
*Mesocletodes elmari* sp. n., CIV male paratype 5. **A** P1 **B** P2 **C** P3 **D** P4. Missing setae indicated by arrows. Scale bars: 50 µm.

**Figure 14.**
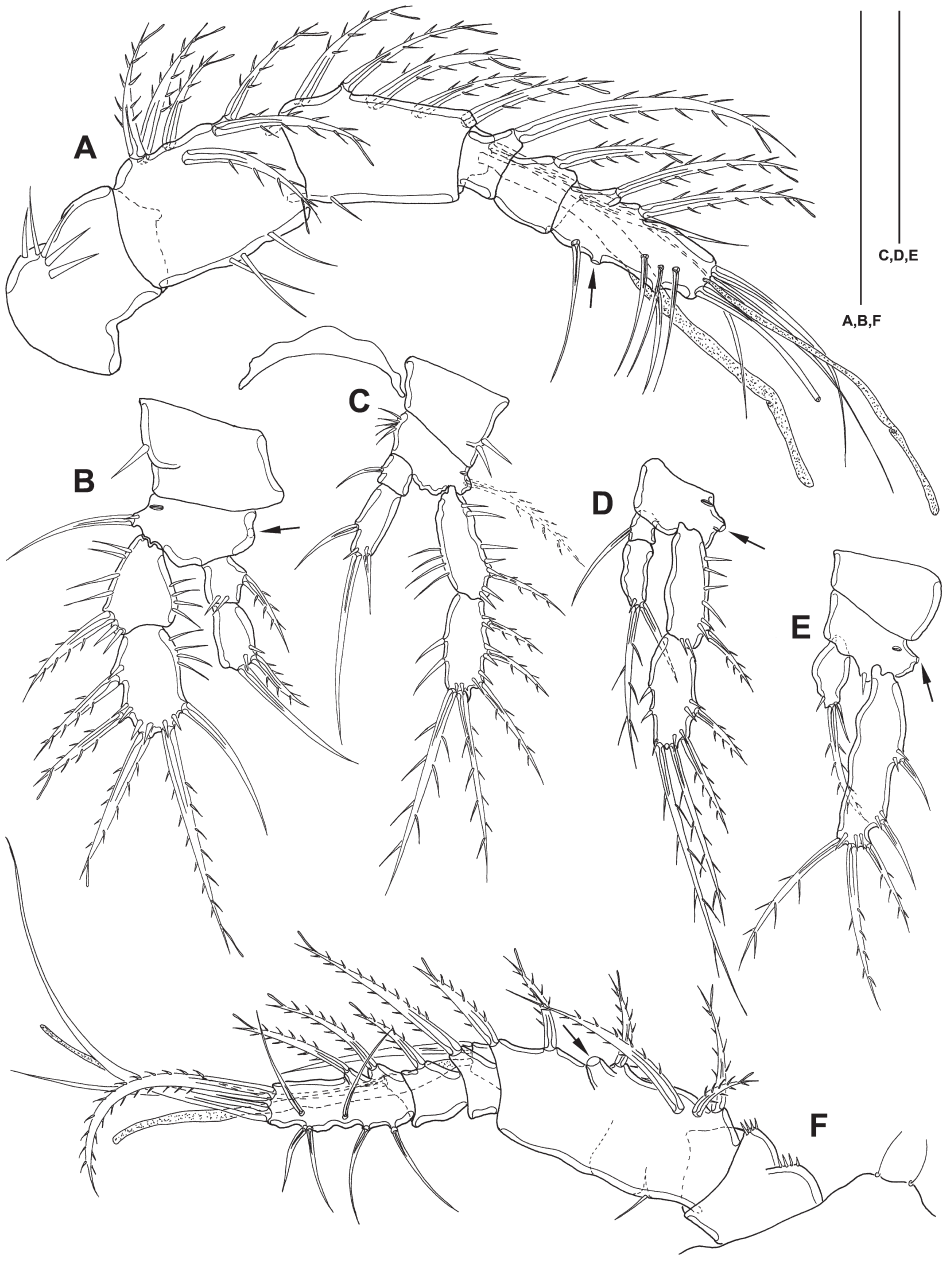
*Mesocletodes elmari* sp. n. **A** CIV male paratype 5, A1 dorsal view **B** CIII paratype 6, P1 **C** CIII paratype 6, P2, outer basal seta supplemented according to counterpart **D** CIII paratype 6, P3 **E** CIII paratype 6, P4 **F** CIII paratype 6, A1. Missing setae indicated by arrows. Scale bars: 50 µm.

(paratypes 3–6) (Figs 8, 10, 12–14) CV female (Fig. 12 C, C1): body length including FR 0.58 mm. Body not of prickly appearance. Penultimate urosomite is not formed. Distal margins of body somites with smooth hyaline frill and, except penultimate one, with sensilla. Extremities A1–P4 (not depicted) as described for adult female but smaller. P5 (Fig. 8 C) exp not separated from benp, setation of exp and basendopodal lobe as in adult female but smaller. P6 (Fig. 8 C1) with 2 setae. GF not expressed.

CV male: body as in CV female. A1 (Fig. 10 B) 6-segmented. Segments 3 and 6 with aes. Second segment large, with a protrusion bearing 1 seta. Setal formula: 1: 0; 2: 8; 3: 9+aes; 4: 2; 5: 2; 6: 9+acrothek (=11+aes). A2–mxp as described for adult female. P1–P4 (not depicted) and P6 (Fig. 8 E1) as described for adult male but smaller. P5 (Fig. 8 E) exp not separated from benp, setation of exp as in adult male but smaller. Right basendopodal lobe with 2 setae and 1 cuticular protrusion, which is missing on the counterpart (see asterisk in Fig. 8 E).

CIV male (Fig. 12 B, B1): body length including FR 0.50 mm. Body not of prickly appearance. 2 penultimate urosomites not formed. Distal margins of body somites with smooth hyaline frill and, except the penultimate one, with sensilla. A1 (Fig. 14 A) 6-segmented. Segments 3 and 6 with aes. Setal formula: 1: 0; 2: 6; 3: 6+aes; 4: 1; 5: 2; 6: 9+acrothek (=11+aes). A2–mxp (not depicted) as described for adult female but smaller P1–P4 (Fig. 13 A–D) with 2-segmented enp and 2-segmented exp. P1–P4 enp2 with 1 inner seta and 1 subterminal, outer seta. For setal formula see Table 2. Setal elements developed as in adult male, P5 (Fig. 8 F) exp not separated from benp. Basendopodal lobe with 2 setae and 1 cuticular protrusion, P5 not fused in the middle. P6 (Fig. 8 F1) with 2 setae. GF not expressed. FR with setular tuft (Fig. 12 B1) close to insertion in telson.

CIII (Fig. 12 A, A1): body length including FR 0.42mm. Body not of prickly appearance. 3 penultimate urosomites not formed. Distal margins of body somites with smooth hyaline frill. A1 (Fig. 14 F) 5-segmented. Setal formula: 1: 0; 2: 8+aes; 3: 1; 4: 2; 5: 9+acrothek (=11+aes). A2–mxp (not depicted) as described for adult female but smaller.

P1–P3 (Fig. 14 B–D) with 2-segmented enp and 2-segmented exp. Exp1 longer than exp2. P4 (Fig. 14 E) exp and enp 1-segmented. For setal formula see Table 2. P5 lost during preparation, P6 not expressed.

##### Morphological variability

(cf. Table 1). The body length including FR is variable: for adult females between 0.57 and 1.06 mm (the majority measured 0.7 to 0.9 mm), for adult males between 0.4 and 0.7 mm, for CV females between 0.5 and 0.75 mm, for CV males between 0.5 and 0.59 mm, for CIV males between 0.4 and 0.64 mm.

The spinulation also seems to be highly variable: the row of spinules ventrally at the telson ranges from numerous, long and slender to few, short and stout. In total, 16 specimens show setular tufts in the FR: six adult females, one CV male and the five CIV males bear setular tufts close to the telson, four adult females close to seta VII. The amount of spinules in A1 segment 3 varies. Four out of 56 adult females, all adult males and copepodid stages possess a non-ornate hyaline frill. A very rare feature (in two adult females, all CIV males) is also the presence of outer setae in P2–P4 enp2 or just in P2 enp2 (one adult female). The number of eggs (2–20) is variable, too.

## Discussion

### Allocation of Mesocletodes elmari sp. n. to Mesocletodes and its position within this genus

Allocation of *Mesocletodes elmari* sp. n. to the taxon *Mesocletodes* is indisputable since all specimens show the apomorphies recognized by Menzel and George (2009): 1) second A1 segment with a strong protrusion bearing 1 strong, bipinnate seta, 2) proximal outer spine of P1 exp3 reduced, 3) spines of P1 exp3 equipped with STE and 4) blades of md gnathobase forming a strong, grinding tooth.

The phylogenetic relationships within *Mesocletodes* are still under discussion. However, a first approach is possible: *Mesocletodes elmari* sp. n. is considered to belong to the “*Mesocletodes inermis* group” as it lacks the characteristic cuticular processes on cephalothorax and telson that are regarded to be autapomorphic to the *Mesocletodes abyssicola*-group (Menzel and George 2009). The extreme elongation of the FR is assumed to be convergent in the new species and the *Mesocletodes abyssicola*-group because several recently observed, but as yet undescribed species of *Mesocletodes* without cuticular processes on cephalothorax and telson also show elongated FR (personal observation). Future investigations, however, will have to prove the phylogenetic relevance of the elongated FR for the *Mesocletodes abyssicola*-group.

*Mesocletodes elmari* sp. n. shows a distinct mxl exopodal segment, and the enp is incorporated into the basis. By contrast, a distinct endopodal segment is described for the mxl of *Mesocletodes bodini* (Soyer 1964; Soyer 1975) and *Mesocletodes irrasus* (T. and A. Scott 1894), whereas the exp is considered to be absent. According to Huys and Boxshall (1991) and Seifried (2003), however, the distinct segments of *Mesocletodes elmari* sp. n., *Mesocletodes bodini* and *Mesocletodes irrasus* are homologous to the exp of other Harpacticoida. The description for *Mesocletodes irrasus* and *Mesocletodes bodini* is therefore erroneous because they show an articulated exp instead of an articulated enp.

### Justification of Mesocletodes elmari sp. n. as a new species

From a morphological point of view *Mesocletodes elmari* sp. n. is similar to *Mesocletodes bodini* and *Mesocletodes parabodini* as these three are the only species of *Mesocletodes* with elongated P1–P4 enp2. *Mesocletodes elmari* sp. n., however, shows clear autapomorphies [plesiomorphic states in brackets] that justify it as a new species:

1) mx seta that is fused to the basis, bears a conspicuously strong spinule-like pinna [seta without spinule-like pinna]

2) P2–P4 exp3 proximal outer seta lost [seta present]

3) P1–P4 enp2 extremely elongated [not elongated]

4) FR strongly elongated between setae III and VII [not elongated]

5) female body of a prickly appearance created by setules that are widened at their bases [no prickly appearance]

6) female P2–P4 enp2 proximal inner seta lost [seta present]

Character 1): The mx seta that is fused to the basiscarries a conspicuously strong spinule-like pinna in *Mesocletodes elmari* sp. n. The corresponding seta in other species of *Mesocletodes* is usually bipinnate with the pinnae of equal size. The loss of all pinnae except one at the anterior side plus the modification of this pinna towards a spinule-like appearance is not recorded for any other species of *Mesocletodes* or Argestidae and is therefore regarded here as derived. This modification thus is considered to be autapomorphic to *Mesocletodes elmari* sp. n.

Character 2): *Mesocletodes elmari* sp. n. lacks the proximal outer seta on P2–P4 exp3. The reduction of outer pereiopodal ornamentation is considered to be derived according to the rule of oligomerization (Huys and Boxshall 1991), but various harpacticoid taxa, including species of Mesocletodes lack this seta convergently. The loss of the proximal outer seta on P2–P4 exp3 is thus considered to be species-specific and therefore autapomorphic to *Mesocletodes elmari* sp. n.

Character 3): Endopodal segments of species of *Mesocletodes* are very short and there are never more than two of them in this genus, many species even have only one single segment. The extreme elongations in P1–P4 enp2 are unique for *Mesocletodes elmari* sp. n. and are considered to be the result of lengthening of the distal endopodal segment. Ontogenetic stages of males do not show a suture that might indicate a fusion of the distal segment with the preceding. Extreme elongations of P1–P4 enp2 are therefore considered here to be autapomorphic to *Mesocletodes elmari* sp. n. A less extreme elongation of these segments, however, occurs also in *Mesocletodes bodini* and *Mesocletodes parabodini*.

Character 4): The FR of *Mesocletodes* are longer than wide, with setae IV, V and VI located terminally, whereas setae I, II, III and VII are located closer to or in the proximal part of the ramus. An extreme elongation between setae III and VII has been discussed as an apomorphy for the *Mesocletodes abyssicola*-group (Menzel and George 2009). However, lacking cuticular processes on cephalotorax and/or telson, *Mesocletodes elmari* sp. n. does not show the other two apomorphies of the *Mesocletodes abyssicola*-group. The extreme elongation of FR thus is considered here to occur convergently in *Mesocletodes elmari* sp. n. and species belonging to the *Mesocletodes abyssicola*-group.

Character 5): Females of *Mesocletodes elmari* sp. n. are characterized by the prickly appearance of the body somites dorsally and laterally. Such coverage is absent in other species of *Mesocletodes* and is therefore regarded here as derived, i.e. an autapomorphic character for *Mesocletodes elmari* sp. n.

Character 6): Endopodal segments do not seem to be fused in *Mesocletodes elmari* sp. n. (see character 3). The proximal inner seta on P2–P4 enp2 in males is considered to be reduced in females. The lack of the proximal inner seta on P2–P4 enp2 is therefore considered here to be autapomorphic to females of *Mesocletodes elmari* sp. n.

### Intraspecific variability in Mesocletodes elmari sp. n.

Intraspecific variability in deep-sea harpacticoids has recently been revealed to be extremely high. For instance, George (2008), Seifried and Martínez Arbizu (2008) as well as Gheerardyn and Veit-Köhler (2009) were able to show that neither setation nor segmentation, nor total length of appendages has to be a reliable character for species discrimination in deep-sea Harpacticoida. Variability in Argestidae has only been recorded for the pereiopodal chaetotaxy of *Argestes angolaensis* George, 2008 (George 2008 and personal observations), and for the shape and number of ventral spinules on the telson in the argestid genus *Eurycletodes* Sars, 1909b (Menzel in press).

For *Mesocletodes* intraspecific variability has not yet explicitly been recorded. However, five species were redescribed at least once, indicating that detected specimens deviate minimally from the type specimen: *Mesocletodes abyssicola* (T. and A. Scott 1901; Sars 1921; Lang 1936), *Mesocletodes bathybia* (Por 1964b; Soyer 1964), *Mesocletodes irrasus* (Scott 1893; T. and A. Scott 1894; Lang 1936; Sars 1909; Soyer 1964) *Mesocletodes monensis* (Thompson 1893; Sars 1921; Lang 1936; Por 1964b;) and *Mesocletodes robustus* (Por 1965; Menzel and George 2009).

Although clear apomorphies were recognized for *Mesocletodes elmari* sp. n., careful morphological examination of the 77 specimens revealed high intraspecific variability (cf. Table 1). The total length of FR, the number and the shape of spinules in various parts of the body, the ornamentation of the hyaline frill and the setation of P2–P4 enp2 is variable. Moreover, few specimens bear setular tufts in various positions on the FR. Setular tufts on the FR near seta VII have only been recorded for *Mesocletodes bodini* (Soyer 1975) and *Mesocletodes parabodini* (Schriever 1983), but corresponding structures near the basis seem to be unique in *Mesocletodes elmari* sp. n. Although setular tufts on the FR seem to be species-specific for *Mesocletodes bodini* and *Mesocletodes parabodini*, the importance of those cuticular structures for species discrimination or even for unraveling phylogenetic relationships remains unclear.

### Sexual dimorphism in Mesocletodes

Many morphological characters of species belonging to *Mesocletodes* are entirely different in both genders. Nevertheless, the identification keys for *Mesocletodes* are exclusively based on the morphology of females (e.g. Wells 2007), possibly due to the fact that merely two males have been described to date. With the aid of these keys, it is nearly impossible to connect a male of *Mesocletodes* to the corresponding female. Consequently the number of species in any deep-sea sample is overestimated, which means faunistic and ecological analyses at the species level are subject to a strong bias. As follows, it appears urgent to quantify the sexually dimorphic modifications in *Mesocletodes*.

**Sexual dimorphism in adults.** The descriptions of *Mesocletodes* contain only females, with the exception of four species: exclusively the male is described for *Mesocletodes angolaensis* and *Mesocletodes fladensis* (the latter description is poorly detailed). Both genders are described for *Mesocletodes faroerensis* and *Mesocletodes thielei*. However, these two species bear a proximal outer spine in P1 exp3 and 3 inner setae on P3 exp3. Moreover, *Mesocletodes faroerensis* bears an inner seta on P1 exp2 and 3 inner setae on P3 exp3, and the md gnathobase of *Mesocletodes thielei* does not form a strong grinding face. Consequently, both species lack autapomorphies of *Mesocletodes* (cf. Menzel and George 2009). Even though the descriptions are poorly detailed and the type material of both species is not available any more, the characters in question are not to be misinterpreted. Thus, *Mesocletodes faroerensis* and *Mesocletodes thielei* have to be excluded from *Mesocletodes*. Future investigations will have to unveil their generic attribution within Argestidae. Consequently, *Mesocletodes elmari* sp. n. is the only known species with matching males and females and therefore convenient for investigations on sexually dimorphic modifications in *Mesocletodes*.

Sexually dimorphic modifications in males of basal Argestidae, such as *Argestes* (George 2008), and *Bodinia* George, 2004 (George 2004) include the A1, P5, P6, and the body size, whereas males of *Mesocletodes* show many more affected characters. The modifications in *Mesocletodes elmari* sp. n. males are comparable to the ones observed in *Mesocletodes angolaensis* and numerous undescribed males from deep-sea samples (personal observation) and are therefore considered to be a good representation of male sexual dimorphism in *Mesocletodes*. 1) The body tapers distally and the setation especially in P1–P4 is very rich and strongly developed in comparison to females. These morphological characters are likely adaptations that help males to stay in the bottom currents once resuspended (cf. characteristics of “typical emergers” [Thistle and Sedlacek 2004; Thistle et al. 2007]) and thus would allow them to explore the sediments for mates. 2) The gut of adult males of *Mesocletodes* is generally empty (personal observation), but the body is filled with several spermatophores instead of food as is reported for several Harpacticoida (cf. Menzel and George 2009; Shimanaga et al. 2009; Wells 1965; Willen 2005). Since the gut of CIV males and CV males of *Mesocletodes elmari* sp. n. is well filled with sediment or detritus, feeding seems to be abandoned at the last molt. It has not been investigated yet whether the gut and digestive tissue are present in adult males. However, the abandonment of feeding and the production of extremely large and numerous spermatophores might be an adaptation to the sparsely populated and oligotrophic deep-sea environments and is therefore considered to represent a derived character state. 3) Mouthparts are either absent, strongly reduced or complete, but apparently not utilized for feeding.Along with the complete reduction of mouthparts, the cephalothorax of *Mesocletodes angolaensis* is slightly depressed in the lateral view and lacks the part that encloses the mouthparts in females. Although the mouthparts of the male of *Mesocletodes elmari* sp. n. do not differ from the female, the ventral edge of the male cephalothorax is less rounded than in the female, but less reduced than in *Mesocletodes angolaensis*.

However, not only the empty gut or the reduction of mouthparts indicates the abandonment of feeding in adult males, but also the A1: most setae on the A1 of the adult male of *Mesocletodes elmari* sp. n. are smooth, merely some in the grasping region of the A1 (segments 3–6) are bipinnate (Fig. 10 A). However, all setae that are smooth in the adult male are strongly pinnate in the two preceding copepodid stages (Figs. 10 B, 14 A). Thus, the loss of pinnae is regarded as another sexually dimorphic modification in adult males since the regression or poorer development of setal elements is typical of non-feeding male copepods (Boxshall and Huys 1998).

Females are generally considered to show the whole character set of a species while the modifications in males are considered to be due to sexual dimorphism (but see George 1998; George 2006a for Ancorabolidae). It is likely, however, that adult females, too, show characters that are connected to the gender because the CV females of *Mesocletodes elmari* sp. n. do not show characters that are typical of adult females: prickly appearance of the body created by setules that are widened at their bases, coxa of P1 externally widened and basal inner seta arising from a prominent protrusion, strongly bent outwards and overlying the enp, P1 enp exceeding exp in length, all extremities bearing conspicuously numerous and strong spinules, and hyaline frill of body somites ornate.

**Sexual dimorphisms in juveniles.** Sexually dimorphic modifications expressed in copepodid stages of *Mesocletodes elmari* sp. n. allow sexing during ontogenetic stages, at least from CV onwards; it is only partially resolved for this species if sexing of CIV is possible because all discovered CIV seem to be of the same gender. A similar constraint applies to the single individual of CIII. This copepodid stage, however, is assumed not to show sexual dimorphism (e.g. Dahms 1990) and is therefore not discussed here.

**Figure 15.**
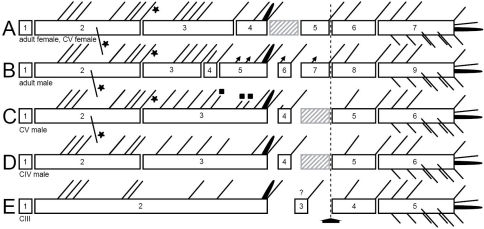
Schematic showing the A1 segmentation and setation of both genders and different copepodid stages of *Mesocletodes elmari* sp. n. **A** adult female and CV female **B** adult male **C** CV male **D** CIV male **E** CIII. Crosshatched segments are considered to be missing or not formed. Solid triangles: sexually dimorphically modified setae, solid squares=setae added at the molt to CV male, solid asterisks=characteristic *Mesocletodes* seta and the subterminal seta in segment 2 in CV and adults. Arrow marks geniculation.

**Sexing of CV.** The male CV and the female CV of *Mesocletodes elmari* sp. n. are distinguishable from the adults by virtue of the overall smaller body size, the lack of the penultimate urosomite and the non-articulated P5 exp. Moreover, the female CV lacks the GF, the male CV lacks the spermatophores and shows strong differences from the adult male in the A1 (Fig. 15 B, C): only six out of nine A1 segments are articulated and several setae are lacking. The position and number of developed setae in these segments, however, resemble the adult male A1 more than the adult female A1 (compare Figs 4 A, 10 A, B, 15 A–C).

**Sexing of CIV.** Careful examination of the A1 and the P5 suggests sexing of the discovered CIV as males.

The five inner setae on the third segment of the CIV A1 (Figs 14 A, 15 D) are almost evenly distributed as is the case in the CV male (Figs 10 B, 15 C). The CV female A1 (cf. Fig. 4 A) has the aes on the fourth segment, while it is on the third in the CV male (Figs 10 B, 15 C). As follows, if the CIV were females, a separation of the aes-bearing segment from the third segment should happen at the next molt. This does not seem plausible, however, because four setae on female segment 3 (Figs 4 A, 15 A) are close to each other in the middle of the segment, the fifth seta inserts distally. An elongation proximally and distally of the evenly distributed four setae in CIV segment 3, plus shortening of the distances between these setae, is not likely. However, an addition of three inner setae at the molt from CIV (Figs 14 A, 15 D) to CV (Figs 10 B, 15 C) in the distal part of this segment (see solid squares in Figs 10 B, 15 C) and maintenance of the distances between the five setae addressed above appear likely. The A1 of the CIV is therefore considered herein to show male characteristics.

The P5 endopodal lobe of the four CIV (Fig. 8 F) has one short, outer seta, one long medial seta and one inner cuticular protrusion, and is therefore in accordance with the CV male (Fig. 8 E). The setation of P5 exp, however, resembles the CV female. Nevertheless, the small depression on the proximal inner edge of the exp (see asterisk in Fig. 8 F) might indicate the emergence of a seta at the next molt, which is only present in males. It is unclear, however, whether harpacticoid CIV show sexually dimorphic modifications in P5 exp. It seems that the CIV of *Mesocletodes elmari* sp. n. do, whereas the opposite is reported for the CIV of an undescribed species of *Orthopsyllus* Brady and Robertson, 1873 (Huys 1990).

P2–P4 enp2 of the discovered CIV bear one inner seta, which is in accordance with female adults and CV. The male adult and CV bear two inner setae in these segments, with the distal seta being homologous to the single seta in the adult female. However, previous studies suggest that endopodal setation is not complete in harpacticoid CIV (Dahms 1990; Dahms 1993; Huys 1990). Thus, the addition of the proximal inner seta at the molt to CV is considered to be likely.

### Ontogenetic development of Mesocletodes elmari sp. n.

Although copepodid stages amount to between 30% and more than 50% of the total deep-sea harpacticoid assemblage, they are excluded from faunistic analyses because confident specific allocation is not possible for many families. For investigations on phylogeny, however, juveniles may be the key to plausible theories (e.g. Ferrari 1988; Fiers 1998; Huys and Boxshall 1991).

Many species descriptions contain short remarks on *Mesocletodes* relationships with other genera and species within the genus. Phylogenetic investigations have been subject to one study to date (Menzel and George 2009), whereas ontogenetic studies on *Mesocletodes* are pending. However, not all copepodid stages of *Mesocletodes elmari* sp. n. are available, and a comparison with juvenile stages of other species of *Mesocletodes* is impossible due to the lack of knowledge. The ontogeny of *Mesocletodes elmari* sp. n. is therefore presented here in a rather descriptive way, but with the purpose to serve as a background for future studies.

A2, mouthparts and FR of Harpacticoida are complete with respect to segmentation and setation from CI onwards (cf. Dahms 1990; Dahms 1992; Dahms 1993). A1 and pereiopods, by contrast, develop gradually by every molt, which is also the case for the habitus: at each molt from CI to adult, one body somite is added anterior to the telson. CV thus shows seven free trunk segments, CIV shows six, and CIII shows five free trunk segments between cephalotorax and telson. Reproductive organs (GF in females and spermatophores in males) are developed at the molt to adult.

**A1.** The female A1 of *Mesocletodes elmari* sp. n. is complete at least at CV, whereas the male A1, which is available from CIV onwards, undergoes extensive modifications at each molt. Segments 3 to 5 of the adult male are part of the third compound segment in CIV males, three setae (marked by solid squares in Figs 10 B, 15 C) are added to this compound segment at the molt to CV. The strongest modifications appear at the molt to adult: the third compound segment is simultaneously separated into segments 3, 4 and 5. Segment 6 of the adult male is distinct at least from CIV onwards, but the proximal seta is added at the molt to CV. Segment 7, directly preceding the geniculation, is not present prior to the molt to adult male.

The characteristic *Mesocletodes* seta (strong, bipinnate, arising from a conspicuous protrusion, see Menzel and George 2009) and a subterminal seta occur at CV in males (compare setae marked by asterisks in Figs 4 A, 10 A, B, 15 A–C). This is likely the case for females, too, as the second A1 segment does not show sexually dimorphic modification regarding the number and position of setae.

Although sexing of the single discovered CIII was impossible, its A1 provides valuable ontogenetic information for *Mesocletodes elmari* sp. n. with respect to the first and the last two A1 segments. These segments, moreover, are not sexually dimorphically modified in CIV or later stages.

Segment 1 lacks a seta at least from CIII onwards (Figs 4 A, 10 A, B, 14 A, F). The presence of a seta on this segment in CI and CII, but the loss at the molt to CIII is discussed to be the case for some harpacticoid species (cf. Boxshall and Huys 1998; Dahms 1989). This, however, could not be followed for *Mesocletodes elmari* sp. n due to the lack of earlier stages than CIII. A similar constraint applies to the development of the last two segments, which are complete at least at CIII (see schematics in Fig. 15), but should also be since CI, as it would be the case in many harpacticoids (cf. Dahms 1989 and references therein).

**P1–P5.** Copepodid development of CI to CV implies extensive changes in P2–P5 with respect to segmentation and setation at each molt. P1 exopodal setation, however, is complete from CI, endopodal setation from CII (Dahms 1993). Changes from the last copepodid stage to adults are restricted to the increase in size (e.g. Dahms 1993; Ferrari 1988). Although earlier stages than CIII have not been found, the investigations on *Mesocletodes elmari* sp. n. are considered to provide an adequate insight into postnaupliar development of P2–P4 in *Mesocletodes* since the progress of the P4 in CIII is comparable to the P2 in CI (Dahms 1993).

Outer elements on the pereiopods of *Mesocletodes elmari* sp. n. occur earlier during ontogeny than inner setae, exps and enps are affected likewise (see P2–P4 of CIII and CIV, Figs 13 B–D, 14 C–E) (cf. Dahms 1993; Ferrari 1988; George 2001). The development of setae in *Mesocletodes elmari* sp. n. is complete at the latest in CIII for P1 (however, it should already be complete in CI, see above), or in CIV for P2–P4 respectively. The separation of the second and third exopodal segments of P1–P4, however, occurs at the molt to CV. P1–P3 endopodal segmentation is complete at the latest in CIII of *Mesocletodes elmari* sp. n., whereas P4 still shows a 1-segmented enp at this stage.

In CIV males the P5 endopodal lobe corresponds to the one in CV and adult, whereas the P5 exp lacks the proximal inner seta (Fig. 8 D, E, F) (see section *Sexual dimorphisms in juveniles*).

On the basis of adult specimens, Menzel and George (2009) recognized four apomorphies for *Mesocletodes* (see above). The above addressed ontogenetic development of *Mesocletodes elmari* sp. n. shows that none of them is characteristic of adults only, but rather appear already during juvenile development.

The characteristic *Mesocletodes* seta on the second A1 segment is developed from CV onwards of both genders. This segment does not show sexually dimorphic modification, except that the setae of females are bipinnate, whereas males bear bare setae. All investigated stages of *Mesocletodes elmari* sp. n. lack the proximal outer spine on P1 exp3. According to Ferrari (1988), this is caused by suppression and further indicates pedomorphosis for this character, i.e. the maintenance of juvenile characters in adults. Considering the harpacticoid pattern of leg development (Dahms 1993; Ferrari 1988), the distal part of the single P1 segment in CI or the second segment in CII–CIV is homologous to the third segment in CV and adult. These parts are fully equipped with all elements characteristic of the third segment. STEs arising from spines on P1 exp3 are only traced from CIII on for *Mesocletodes elmari* sp. n. However, it seems likely that these extensions exist from CI as the setae they are associated with do so. The same applies to the strong grinding tooth at the md gnathobase. This is developed at least at CIII of *Mesocletodes elmari* sp. n., but according to Dahms (1990), for example, this should be the case from CI onwards.

### Brief remarks on the geographic distribution of Mesocletodes elmari sp. n.

Various taxa of benthic harpacticoid copepods show distribution ranges at the species level that extend over thousands of kilometers across Atlantic, Southern Ocean and Pacific abyssal plains: Ancorabolidae Sars, 1909a (George 2006b; Gheerardyn and George 2010), Argestidae (Menzel and George 2009; Menzel in press), Canthocamptidae Sars, 1906 (Mahatma 2009), Ectinosomatidae Sars, 1903 (Seifried and Martínez Arbizu 2008), Paramesochridae Lang, 1944 (Gheerardyn and Veit-Köhler 2009; Plum and George 2009).

In the case of *Mesocletodes*, as well, the sampling localities known up to now suggest an extremely wide distribution of this genus: the North Atlantic (Scandinavian coast [Lang 1948; Pesta 1927; Por 1964a; Por 1965; Sars 1909; Sars 1921], Irish, English and Scottish coasts [T. Scott, 1900; T. Scott, 1906; Thompson 1893; Wells 1965], Porcupine Abyssal Plain [Gheerardyn 2007; Gheerardyn et al. 2010], Spitzbergen coast [Lang 1936], Arctic Ocean [T. and A. Scott 1901; Smirnov 1946], Icelandic coast and Iceland Faroe Ridge [Schriever 1983; Schriever 1985], Greenlandic coast [Jespersen 1939], off North Carolina [Coull 1973] Nova Scotia Rise [Thistle and Eckman 1990], French Atlantic coast [Bodin 1968], Iberian Basin [Becker et al. 1979], Great Meteor Bank [George and Schminke 2002]), the Mediterranean Sea (Guidi-Guilvard et al. 2009; Por 1964b; Soyer 1964; Soyer 1975), the Red Sea (Por 1967), the Pacific Ocean (Peru Trench [Becker et al. 1979], off Hawaii [Mahatma 2009], off the Californian coast [Thistle et al. 2007], off the Japanese coast [Shimanaga et al. 2004]), the Indian Ocean (Por 1986a), the South Atlantic Ocean (Southwest Atlantic [George 2005], the Southeast Atlantic [Menzel and George 2009]). However, the distribution of *Mesocletodes* at the species level has been addressed briefly (Menzel and George 2009), and is subject to ongoing studies.

The record of *Mesocletodes elmari* sp. n. in the North Atlantic Ocean and South Atlantic Ocean, the Southern Ocean, the Pacific Ocean and the South Indian Ocean extends the knowledge on the distribution of *Mesocletodes* and points a worldwide distribution at the species level. Future studies will have to deal with the means of dispersal as well as ecological and biological needs of species belonging to *Mesocletodes* to help explain the distributional patterns.

## Supplementary Material

XML Treatment for Mesocletodes

## References

[B1] Balzer W Alheit J Emeis K-C Lass HU Türkay M (2006) Southeast Atlantic. Cruise No. 48 METEOR-Berichte 06–5. Hamburg: Leitstelle METEOR, Institut für Meereskunde der Universität Hamburg 1–219.

[B2] Barnett PRO Watson J Connelly D (1984) A multiple corer for taking virtually undisturbed samples from shelf, bathyal and abyssal sediments. Oceanologica Acta 7:399-408.

[B3] Becker K-H Noodt W Schriever G (1979) Eidonomie und Taxonomie abyssaler Harpacticoidea (Crustacea, Copepoda) Teil 2. Paramesochridae, Cylindropsyllidae und Cletodidae. Meteor Forschungsergebnisse Reihe D - Biologie Supplement: 1–37.

[B4] Bodin P (1968) Copépodes Harpacticoïdes des étages bathyal et abyssal du Golfe de Gascogne. Mémoires du muséum national d’histoire naturelle 55:1-107.

[B5] Boxshall G Huys R (1998) The ontogeny and phylogeny of copepod antennules. Philosophical Transactions of the Royal Society of London, Series B 353:765-786. 10.1098/rstb.1998.0242

[B6] Boxshall GA Halsey S (2004) An Introduction to Copepod Diversity. Andover: Ray Society. 2000.

[B7] Brady GS Robertson D (1873) Contributions to the Study of the Entomostraca. No. VIII. On Marine Copepoda taken in the West of Ireland. Annals of Natural History 4:126-142.

[B8] Coull BC (1973) Meiobenthic harpacticoida (crustacea, copepoda) from the deep sea off North Carolina. IV. the families Cletodidae T. Scott and Ancorabolidae Sars. Transactions of the American Microscopical Society 92:604-620. 10.2307/3225271

[B9] Dahms H-U (1989) Antennule development during copepodite phase of some representatives of Harpacticoida (Crustacea, Copepoda). Bijdragen tot de Dierkunde 59:159-189.

[B10] Dahms H-U (1990) The first nauplius and the copepodite stages of *Thalestris longimana* Claus, 1863 (Copepoda, Harpacticoida, Thalestridae) and their bearing on the reconstruction of phylogenetic relationships. Hydrobiologia 202:33-60.

[B11] Dahms H-U (1992) Metamorphosis between naupliar and copepodid phases in the Harpacticoida. Philosophical Transactions of the Royal Society of London Series B- Biological Sciences 335:221-236. 10.1098/rstb.1992.0020

[B12] Dahms H-U (1993) Copepodid development in Harpacticoida (Crustacea, Copepoda). Microfauna Marina 8:195-245.

[B13] Fahrbach E (2006) The Expedition ANTARKTIS-XXII/3 of the Research Vessel “Polarstern” in 2005. Berichte zur Polar- und Meeresforschung 533:1-246.

[B14] Ferrari FD (1988) Developmental patterns in numbers of ramal segments of Copepod post maxillipedal legs. Crustaceana 54:256-293.doi:10.1163/156854088X00168

[B15] Fiers F (1998) Female leg 4 development in Laophontidae (Harpacticoida): a juvenile adaptation to precopulatory behaviour. Journal of Marine Systems 15:41-51. 10.1016/S0924-7963(97)00050-X

[B16] Fütterer DK Brandt A Poore GCB (2003) The Expeditions ANTARKTIS-XIX/3-4 of the Research Vessel POLARSTERN in 2002. Berichte zur Polar- und Meeresforschung 470:1-174.

[B17] Galéron J Fabri M-C (2004) Rapport de Campagne. Nodinaut 17 Mai-28 Juin 2004, Direction des Recherches Océaniques, Département Environnement Profond, IFREMER 1–177.

[B18] George KH (1998) *Polyascophorus*, a new genus of Ancorabolidae (Crustacea, Copepoda), including the description of two new species and the re-allocation of *Ceratonotus gorbunovi*. Vie et Milieu 48:141-155.

[B19] George KH (2001) First record of the “genus” *Ancorabolus* Norman 1903 from the Southern Hemisphere, including analyses of copepodid development (Crustacea, Copepoda, Harpacticoida, Ancorabolidae). Senckenbergiana Maritima 81:23-36.

[B20] George KH (2004) Description of two new species of *Bodinia*, a new genus incertae sedis in Argestidae Por, 1986 (Copepoda, Harpacticoida), with reflections on argestid colonization of the Great Meteor Seamount plateau. Organisms, Diversity & Evolution 4: 241–264. 10.1016/j.ode.2004.02.003

[B21] George KH (2005) Sublittoral and bathyal Harpacticoida (Crustacea, Copepoda) of the Magellan region. Composition, distribution and species diversity of selected major taxa. Scientia Marina 69:147-158.

[B22] George KH (2006a) New *Ancorabolinae* Sars, 1909 (Copepoda: Harpacticoida: Ancorabolidae) of the Atlantic and the Pacific Ocean. The taxa *Ceratonotus* Sars, and *Dendropsyllus* Conroy-Dalton. Meiofauna Marina 15:87-122.

[B23] George KH (2006b) *Ancorabolinae* Sars (Copepoda: Harpacticoida: Ancorabolidae) of the deep Atlantic Ocean. *Ancorabolina chimaera* gen. et sp. n. including remarks to ancorabolid phylogeny and to the evolution of the first natatorial leg in comparison with Laophontoidea T. Scott. Meiofauna Marina 15:157-176.

[B24] George KH (2008) *Argestes angolaensis* sp. n. (Copepoda: Harpacticoida: Argestidae) from the Angola Basin (Southeast Atlantic), and the phylogenetic characterization of the taxon *Argestes* Sars, including the redescription of *A. mollis* Sars, 1910, and *A. reductus* (Itô, 1983). Zootaxa 1866:223-262.

[B25] George KH Schminke HK (2002) Harpacticoida (Crustacea, Copepoda) of the Great Meteor Seamount, with first conclusions as to the origin of the plateau fauna. Marine Biology 144: 887–895. 10.1007/s00227-002-0878-6

[B26] Gheerardyn H (2007) Biodiversiteit en taxonomie van harpacticoide copepoden geassocieerd met koraalsubstraten van tropen en diepzee. PhD, Universiteit Ghent.

[B27] Gheerardyn H Veit-Köhler G (2009) Diversity of large-scale biogeography of Paramesochridae (Copepoda, Harpacticoida) in South Atlantic abyssal plains and the deep Southern Ocean. Deep-Sea Research I 56: 1804–1815. 10.1016/j.dsr.2009.05.002

[B28] Gheerardyn H De Troch M Vincx M Vanreusel A (2010) Diversity and community structure of harpacticoid copepods associated with cold-water coral substrates in the Porcupine Seabight (North-East Atlantic). Helgoland Marine Research 64: 53–62. doi:10.1007/s10152-009-0166-7

[B29] Gheerardyn H George KH (2010) New representatives of the genus *Ancorabolina* George, 2006 (Copepoda, Harpacticoida, Ancorabolidae) including remarks on ancorabolid phylogeny. Zoological Journal of the Linnean Society 158: 16–55. 10.1111/j.1096-3642.2009.00567.x

[B30] Guidi-Guilvard LD Thistle D Khripounoff A Gasparini S (2009) Dynamics of benthic copepods and other meiofauna in the benthic boundary layer of the deep NW Mediterranean Sea. Marine Ecology Progress Series 396: 181–195. 10.3354/meps08408

[B31] Huys R (1990) A new family of harpacticoid copepods and an analysis of the phylogenetic relationships within the Laophontoidea T. Scott. Bijdragen tot de Dierkunde 60:79-120.

[B32] Huys R Boxshall G (1991) Copepod evolution. The Ray Society: London.

[B33] Huys R (1996) Superornatiremidae fam. nov. (Copepoda: Harpacticoida): an enigmatic family from North Atlantic anchihaline caves. Scientia Marina 60:497-542.

[B34] Jespersen P (1939) The zoology of east Greenland. Copepods. Meddelelse Grønland 121:1-66.

[B35] Kalogeropoulou V Bett BJ Gooday AJ Lampadariou N Martínez Arbizu P Vanreusel A (2010) Temporal changes (1989–1999) in deep-sea metazoan meiofaunal assemblages on the Porcupine Abyssal Plain, NE Atlantic. Deep-Sea Research II 57: 1383–1395. 10.1016/j.dsr2.2009.02.002

[B36] Lang K (1936) Die während der schwedischen Expedition nach Spitzbergen 1898 und nach Grönland 1899 eingesammelten Harpacticiden. Kungliga Svenska Vetenskapsakademiens Handlingar 15:1-55.

[B37] Lang K (1944) Monographie der Harpacticiden (Vorläufige Mitteilung). Almqvist & Wiksells, Uppsala.

[B38] Lang K (1948) Monographie der Harpacticiden I+II, reprint. Otto Koeltz Science Publishers: Königstein.

[B39] Lang K (1965) Copepoda Harpacticoida from the Californian Pacific coast. Kungliga Svenska Vetenskapsakademiens Handlingar 10:1-560.

[B40] Mahatma R (2009) Meiofauna Communities of the Pacific Nodule Province: abundance, diversity and community structure. Unpublished PhD, Carl von Ossietzky Universität Oldenburg.

[B41] Martínez Arbizu P Schminke HK (2005) DIVA-1 expedition to the deep sea of the Angola Basin in 2000 and DIVA-1 workshop in 2003. Organisms, Diversity & Evolution 5:1-2. 10.1016/j.ode.2004.11.009

[B42] Menzel L (in press) A new species of *Eurycletodes* Sars, 1909 (Copepoda, Harpacticoida, Argestidae) from the Southern hemisphere including remarks on the phylogeny of and within this genus. Helgoland Marine Research. 10.1007/s10152-010-0237-9

[B43] Menzel L George KH (2009) Description of four new species of *Mesocletodes* Sars, 1909 (Copepoda, Harpacticoida, Argestidae) and redescription of *Mesocletodes robustus* Por, 1965 from the South Atlantic, including remarks on the *Mesocletodes abyssicola*-group. Zootaxa 2096:214-256.

[B44] Michels J Büntzow M (2010) Assessment of Congo red as fluorescence marker for the exoskeleton of small crustaceans and the cuticle of polychaetes. Journal of Microscopy 238: 95–101. 10.1111/j.1365-2818.2009.03360.x 20529057

[B45] Noodt W (1952) Marine Harpacticiden (Cop.) aus dem eulitoralen Sandstrand der Insel Sylt. Abhandlungen der mathematisch naturwissenschaftlichen Klasse 3:105-142.

[B46] Pesta O (1927) Copepoda non parasitica. Die Tierwelt der Nord- und Ostsee. Akademische Verlagsgesellschaft, Leipzig

[B47] Pfannkuche O Müller TJ Nellen W Wefer G (2000) Ostatlantik. Cruise No. 42 METEOR-Berichte 00-1. Hamburg: Leitstelle METEOR, Institut für Meereskunde der Universität Hamburg, 1–259.

[B48] Plum C George KH (2009) The paramesochrid fauna of the Great Meteor Seamount (Northeast Atlantic) including the description of a new species of *Scottopsyllus (Intermedopsyllus)* Kunz (Copepoda: Harpacticoida: Paramesochridae). Marine Biodiversity. 10.1007/s12526-009-0022-7

[B49] Pollard RT Sanders R (2006) RRS Discovery Cruises 285/286, 3 Nov – 10 Dec 2004; 13 Dec 2004 – 21 Jan 2005. CROZet circulation, iron fertilization and EXport production experiment (CROZEX) Cruise ReportSouthampton: Southampton Oceanography Centre 260.

[B50] Por FD (1964a) Les Harpacticoïdes (Crustacea, Copepoda) des fonds meubles du Skagerak. Cahiers de Biologie Marine 5:233-270.

[B51] Por FD (1964b) A study of the Levatine and Pontic Harpacticoida (Crustacea, Copepeoda). Zoologische Verhandelingen 64:1-128.

[B52] Por FD (1965) Harpacticoida (Crustacea, Copepoda) from muddy bottoms near Bergen. Sarsia 21:1-16.

[B53] Por FD (1967) Level Bottom Harpacticoida (Crustacea, Copepoda) from Eilat (Red Sea), Part I. Israel Journal of Zoology 16:101-165.

[B54] Por FD (1986a) A re-evaluation of the family Cletodidae Sars, Lang (Copepoda, Harpacticoida). Syllogeus 58:419-425.

[B55] Por FD (1986b) New deep sea Harpacticoidea (Copepoda) of cletodid type, collected in the Indian Ocean by R/V «Anton Bruun» in 1964. Crustaceana 50:78-98. 10.1163/156854085X00099

[B56] Rose A Seifried S Willen E George KH Veit-Köhler G Bröhldick K Drewes J Moura G Martínez Arbizu P Schminke HK (2005) A method for comparing within-core alpha diversity values from repeated multicorer samplings, shown for abyssal Harpacticoida (Crustacea: Copepoda) from the Angola Basin. Organisms, Diversity & Evolution 5:3-17. 10.1016/j.ode.2004.10.001

[B57] Sars GO (1903) Copepoda Harpacticoida. Parts I & II. Misophrioida, Longipediidae, Cerviniidae, Ectinosomatidae (part). An Account of the Crustacea of Norway, with short descriptions and figures of all the species 5:1-28.

[B58] Sars GO (1906) Copepoda Harpacticoida. Parts XV & XVI. Diosaccidae (concluded), Canthocamptidae (part). An Account of the Crustacea of Norway, with short descriptions and figures of all the species 5:173-196.

[B59] Sars GO (1909a) Copepoda Harpacticoida. Parts XXVII & XXVIII. Cletodidae (concluded), Anchorabolidae, Cylindropsyllidae, Tachidiidae (part). An Account of the Crustacea of Norway, with short descriptions and figures of all the species 5:305-336.

[B60] Sars GO (1909b) Copepoda Harpacticoida. Parts XXV & XXVI. Laophontidae (concluded), Cletodidae (part). An Account of the Crustacea of Norway with short descriptions and figures of all the species 5:277-304.

[B61] Sars GO (1910) Copepoda Harpacticoida. Parts XXIX & XXX. Tachidiidae (concluded), Metidae, Balaenophilidae, Supplement (part). An Account of the Crustacea of Norway with short descriptions and figures of all the species 5:337-368.

[B62] Sars GO (1921) Copepoda Supplement. An Account of the Crustacea of Norway with short descriptions and figures of all the species 7:1-121.

[B63] Schriever G (1983) New Harpacticoida (Crustacea, Copepoda) from the north Atlantic Ocean. III. New species of the family Cletodidae. Meteor Forschungsergebnisse D Supplement: 65–83.

[B64] Schriever G (1985) New Harpacticoida from the north Atlantic Ocean. VII The description of five new species of the genus *Mesocletodes* Sars (Cletodidae). Mitteilungen aus dem Zoologischen Museum der Universität Kiel 2:1-12.

[B65] Scott T (1893) Additions to the fauna of the Firth of Forth. 11th Annual Report of the Fishery Board for Scotland for the Year 1892 5:197-219.

[B66] Scott T Scott A (1894) On some new and rare Crustacea from Scotland. Annals and Magazine of Natural History 13: 141.

[B67] Scott T (1900) III. Notes on gatherings of crustacea, collected for the most part by the fishery steamer »Garland» and the steam trawler »St. Andrew» of Aberdeen, and examined during the year 1900. Annual Report of the Fishery Board of Scotland 19.

[B68] Scott T Scott A (1901) On some new Entomostraca collected in the Arctic Seas by W. Bruce. Annals and Magazine of Natural History 8: 347.

[B69] Scott T (1906) A catalogue of land, fresh-water, and marine crustacea found in the basin of the river Forth and its estuary. Part II. The Ostracoda, Copepoda, and Cirripedia. Proceedings of the Royal Physical Society of Edinburgh 16:267-386.

[B70] Seifried S (2003) Phylogeny of Harpacticoida (Copepoda): Revision of “Maxillipedasphalea” and Exanechentera. Cuvillier Verlag: Göttingen.

[B71] Seifried S Martínez Arbizu P (2008) A new and exceptional species of *Bradya* Boeck, 1873 (Copepoda: Harpacticoida: Ectinosomatidae) from the abyssal plain of the Angola Basin and the variability of deep-sea Harpacticoida. Zootaxa 1866:303-322.

[B72] Seifried S Veit-Köhler G (2010) Redescription of *Bradya typica* Boeck, 1873 (Copepoda: Harpacticoida: Ectinosomatidae) with the first description of the male. Helgoland Marine Research 64: 1–20. 10.1007/s10152-009-0165-8

[B73] Shimanaga M Shirayama Y (2003) Sex ratio and reproductive activity of benthic copepods in bathyal Sagami Bay (1430 m), central Japan. Progress in Oceanography 57: 97–107. 10.1016/S0079-6611(03)00053-3

[B74] Shimanaga M Kitazato H Shirayama Y (2004) Temporal patterns in diversity and species composition of deep-sea benthic copepods in bathyal Sagami Bay, central Japan. Marine Biology 144: 1097–1110. 10.1007/s00227-003-1273-7

[B75] Shimanaga M Lee W Nomaki H Iijima K (2009) Sex ratio and gut contents of the deep-sea Harpacticoid *Neocervinia itoi* and other Cerviniids: a possibility of reduced foraging among males. Journal of Crustacean Biology 29: 182–191. 10.1651/08-3036.1

[B76] Smirnov SS (1946) New species of Copepoda-Harpacticoida from the northern Arctic Ocean. Trudy Dreifuyushchei Ekspeditsyai Glausemov Ledokol Por “Sedov” 3:231-263.

[B77] Soyer J (1964) Copépodes harpacticoïdes de I’étage bathyal de la région de Banyuls-sur-Mer. V. Cletodidae. T. Scott. Vie et Milieu 15:573-643.

[B78] Soyer J (1975) Contribution à l’étude des Copépodes harpacticoïdes de méditerrannée occidentale 13. le genre *Mesocletodes* Sars (Cletodidae T. Scott) systématique, écologie. Vie et Milieu 25:157-174.

[B79] Thistle D Eckman JE (1990) What is the sex ratio of harpacticoid copepods in the deep sea? Marine Biology 107: 443–447. 10.1007/BF01313427

[B80] Thistle D Sedlacek L (2004) Emergent and non-emergent species of harpacticoid copepods can be recognized morphologically. Marine Ecology Progress Series 266:195-200. 10.3354/meps266195

[B81] Thistle D Sedlacek L Carman KR Fleeger JW Barry JP (2007) Emergence in the deep sea: Evidence from harpacticoid copepods. Deep Sea Research (Part I, Oceanographic Research Papers) 54: 1008–1014. 10.1016/j.dsr.2007.03.002

[B82] Thompson IC (1893) Revised report on the Copepoda of Liverpool Bay. Proceedings and Transactions of the Liverpool Biological Society 7:175-230.

[B83] Türkay M Pätzold J (2009) Southwestern Indian Ocean-Eastern Atlantic Ocean. Cruise No. 63 METEOR-Berichte 09–3. Hamburg: Leitstelle METEOR, Institut für Meereskunde der Universität Hamburg 1–98.

[B84] Vasconcelos DM Veit-Köhler G Drewes J Perreira dos Santos PJ (2009) First record of the genus *Kliopsyllus* Kunz, 1962 (Copepoda Harpacticoida, Paramesochridae) from Northeastern Brazil with description of the deep-sea species *Kliopsyllus minor* sp. n. Zootaxa 2096:327-337.

[B85] Wells JBJ (1965) Copepoda (Crustacea) from the meiobenthos of some Scottish marine sub-littoral muds. Proceedings of the Royal Society of Edinburgh, Section B, Biological Sciences 69:1-33.

[B86] Wells JBJ (2007) An annotated checklist and keys to the species of Copepoda Harpacticoida (Crustacea). Zootaxa 1568:1-872.

[B87] Willen E (2005) A new species of *Paranannopus* Lang, 1936 (Copepoda, Harpacticoida, Pseudotachidiidae) with atrophic mouthparts from the abyssal of the Angola Basin. Organisms, Diversity & Evolution 5: 19–27. 10.1016/j.ode.2004.10.002

[B88] Willen E (2006) A new species of Copepoda Harpacticoida, *Xylora calyptogenae* spec. n., with a carnivorous life-style from a hydrothermally active submarine volcano in the New Ireland Fore-Arc system (Papua New Guinea) with notes on the systematics of the *Donsiellinae* Lang, 1948. Helgoland Marine Research 60: 257–272. 10.1007/s10152-006-0040-9

[B89] Willen E (2009) *Nyxis rostrocularis*, a new genus and species of Paranannopinae Por, 1986 (Copepoda, Harpacticoida) from the Southern Atlantic deep sea. Zootaxa 2096:299-312.

[B90] Willen E Dittmar J (2009) A new genus of Pseudomesochrinae Willen, 1996 (Copepoda, Harpacticoida, Pseudotachidiidae) from the Guinea Basin. Zootaxa 2096:287-298.

